# Antimicrobial resistance among Gram-positive agents of bacteraemia in the UK and Ireland: trends from 2001 to 2019

**DOI:** 10.1093/jac/dkaf249

**Published:** 2025-10-27

**Authors:** Rosy Reynolds, Shazad Mushtaq, Russell Hope, Carolyne Horner, Aiysha Chaudhry, Rachael Adkin, Olisaeloka Nsonwu, Michael Allen, Christopher Longshaw, Benjamin J Parcell, David M Livermore

**Affiliations:** Population Health Sciences, University of Bristol, Bristol BS8 2PS, UK; British Society for Antimicrobial Chemotherapy, 53 Regent Place, Birmingham B1 3NJ, UK; Antimicrobial Resistance and Healthcare Associated Infections Reference Unit, UK Health Security Agency, Colindale, London NW9 5EQ, UK; Antimicrobial Resistance and Healthcare Associated Infections Division, UK Health Security Agency, Colindale, London NW9 5EQ, UK; British Society for Antimicrobial Chemotherapy, 53 Regent Place, Birmingham B1 3NJ, UK; Antimicrobial Resistance and Healthcare Associated Infections Reference Unit, UK Health Security Agency, Colindale, London NW9 5EQ, UK; Antimicrobial Resistance and Healthcare Associated Infections Reference Unit, UK Health Security Agency, Colindale, London NW9 5EQ, UK; Antimicrobial Resistance and Healthcare Associated Infections Division, UK Health Security Agency, Colindale, London NW9 5EQ, UK; British Society for Antimicrobial Chemotherapy, 53 Regent Place, Birmingham B1 3NJ, UK; Medical Affairs, MSD (UK) Limited, 120 Moorgate, London EC2M 6UR, UK; British Society for Antimicrobial Chemotherapy, 53 Regent Place, Birmingham B1 3NJ, UK; Scientific Affairs, Shionogi B.V., Fifty Paddington, 50 Eastbourne Terrace, Paddington W2 6LG, UK; Division of Population Health and Genomics, School of Medicine, University of Dundee, Ninewells Hospital and Medical School, Dundee DD1 9SY, UK; Department of Medical Microbiology, Ninewells Hospital and Medical School, Dundee DD1 9SY, UK; Antimicrobial Resistance and Healthcare Associated Infections Reference Unit, UK Health Security Agency, Colindale, London NW9 5EQ, UK; Norwich Medical School, University of East Anglia, Norwich NR4 7TJ, UK

## Abstract

**Objectives:**

The BSAC Bacteraemia Resistance Surveillance collected isolates from UK and Irish hospitals for central testing. Concurrent UKHSA surveillance collated English hospitals’ own susceptibility data. Results were collated and compared.

**Methods:**

BSAC Surveillance collected quotas of isolates per site annually from 2001 to 2019. MIC testing was by BSAC agar dilution, with resistance mechanisms identified by synergy tests, interpretive reading and PCR. The UKHSA sought hospitals’ data on all bacteraemia isolates.

**Results:**

Both surveillance systems recorded dramatic falls in MRSA, from *c.* 40% of bloodstream *Staphylococcus aureus* in 2001 to <10% by 2019. Both noted rises in the proportion of MRSA (especially) and MSSA resistant to fusidic acid, along with declines of ciprofloxacin and macrolide resistance amongst MRSA. Methicillin resistance also fell among coagulase-negative staphylococci, albeit only modestly; fusidic acid resistance rose. Shifts for pneumococci were complex, reflecting vaccine-contingent serotype displacements; resistance rates remained low, with high-dose penicillin almost universally active. *Enterococcus faecium* became more prevalent relative to *Enterococcus faecalis*; vancomycin resistance averaged 29% among *E. faecium* versus 2% in *E. faecalis*, without trend. Erythromycin resistance rose among groups B, C and G (but not group A) streptococci. Oxazolidinones, tigecycline, daptomycin and anti-PBP2′ cephalosporins retained near-universal activity against target species, except that tigecycline has been compromised by breakpoint reductions for streptococci.

**Conclusions:**

Gram-positive pathogens were the dominant historical pathogens of bacteraemia. The trends seen here—with many near-universally active antibiotics—indicate little hazard of this situation returning. Nevertheless, few treatments exist in some settings, notably multi-resistant *E. faecium* endocarditis.

## Introduction

Gram-positive pathogens cause *c.* 50% of bacteraemias in England.^1,2^ Staphylococcal bacteraemias often are vascular-access-device-associated, or follow soft tissue infections. Pneumococcal bacteraemias arise by overspill from pneumonia. Enterococcal bacteraemias mostly occur in patients with severe underlying disease, as do bacteraemias involving α- and non-haemolytic streptococci. β-Haemolytic streptococci remain important too: *Streptococcus pyogenes* (‘Group A Streptococcus’) is an aggressive pathogen which, before antibiotics, caused most bacteraemias^3^; *Streptococcus agalactiae* (‘Group B Streptococcus’) is a vaginal colonist associated with neonatal sepsis.

The past 20 years have seen major shifts. In the UK, MRSA bacteraemias declined following re-emphasis of infection prevention and control from 2001^4^; MSSA bacteraemias declined far less and have rebounded.^1,5^ Since 2006, pneumococcal infections have been disrupted by conjugate vaccines (PCVs), reducing bacteraemias in vaccinated children and, by a herd effect, in adults.^6,7^  *Enterococcus faecium* has become increasingly prevalent compared with *Enterococcus faecalis*.^8^

Anti-Gram-positive antibiotics introduced since 2000 include lipoglycopeptides, oxazolidinones, glycyl- and fluorinated tetracyclines, lipopeptides and PBP2′-targetted cephalosporins. There are now around 16 drugs in nine classes reliably active against MRSA, compared with three (vancomycin, teicoplanin and minocycline) in two classes in 1997.^9^ Caveats are: (i) we still lack new bactericidal options for multi-resistant *E. faecium* endocarditis and (ii) resistance has been seen to every newer agent.^10^

From 2001 to 2019 the BSAC Bacteraemia Resistance Surveillance Programme collected Gram-positive pathogens from bacteraemia for centralized testing, with molecular investigation as appropriate.^11^ We present the results, comparing observed trends with those recorded in UKHSA bacteraemia surveillance, which collates hospital laboratories’ own susceptibility data.

## Materials and methods

Methods for the BSAC and UKHSA surveillances are described elsewhere.^11^ Briefly, the BSAC Programme sought up to 250 isolates per species group annually from 25 laboratories, increasing to 280 from 40 sites in 2010–15; actual numbers of contributing laboratories typically were slightly smaller, reflecting a few sites that were recruited but failed to collect. For *S. aureus* the target doubled to 500 or 560 isolates from 2008 onwards.

Bacterial identification was initially by classical methods, increasingly replaced by MALDI-TOF. MICs were determined by BSAC agar dilution; *mecA* and *mupA* were sought by PCR; pneumococcal serotypes were determined by classical methods or, latterly, inferred from WGS.^11^ The antibiotics tested included core agents tested in all years under the aegis of the BSAC as well as those included for variable periods contingent on sponsorship by funders. All these aspects are generic to the data papers of this Supplement and are fully described elsewhere.^11^

Tables S1–S4 (available as Supplementary data at *JAC* Online) detail numbers of laboratories contributing isolates annually, also necessary data amendments and exclusions. Tables S5–S10 detail breakpoints (EUCAST v12.0, 2022) and susceptibility tests by organism, antimicrobial and years included; not all antimicrobials were tested every year. Tables S11–S14 and Figures S1–S3 cover patient characteristics, noting any missing data. MIC distributions are also presented.

### Analysis

Analysis was descriptive and largely graphical, using Stata 18.0 (StataCorp LLC: College Station, TX) and Bischoff’s colour vision-sensitive ‘plotplainblind’ graph scheme.^12^ Missing data were excluded in the calculation of percentages.

## Results

### BSAC isolate collection

The collection comprised 7554 *S. aureus*, with 3494 to 4486 isolates for each of the other groups (Table S2). The proportion from male patients varied from 52% for β-haemolytic streptococci to 63% for *S. aureus* (see Table S11, which also provides detail by species and, for staphylococci, by methicillin resistance status). The median age was lowest for CoNS (55 years) and highest for enterococci (68 years); proportions from patients aged ≥80 years ranged from 7% (CoNS) to 23% (*Streptococcus pneumoniae*) (Table S12 and Figure S1). All collection groups included subgroups of isolates from infants <1 year old (Table S12). These were largest for CoNS (11% overall; 31% for *Staphylococcus capitis*) and β-haemolytic streptococci (11% overall; 29% for group B). The proportion of ‘hospital-acquired’ isolates, from patients hospitalized for >48 h, declined over time but was consistently greatest for CoNS and enterococci (which also were the two groups with the highest proportions of ICU samples) and least for *S. pneumoniae* and β-haemolytic streptococci (Table S13 and Figures S2 and S3). Data for sources of infection were limited to 2001–13 and were incompletely reported by Collecting Laboratories; they suggested that the proportion of device-associated isolates fell over time—substantially for *S. aureus* and enterococci and less so for CoNS (Table S14).

### UKHSA data extract

Tables S15–S16 and Figures S4–S5 show the increasing numbers of bacteraemia reports for most organism groups over time and an increasing proportion with susceptibility data. In the period analysed for comparison with BSAC data (2001–2019) numbers of reports for organism groups except CoNS ranged from 1857/year (α-haemolytic streptococci, 2001) to 14 112 (*S. aureus*, 2003). CoNS reports rose dramatically—from 5402 (9.1% of all bacteraemia reports) in 2001 to 17 032 in 2014 (16.1%), and 41 875 in 2019 (24.0%). These shifts, at least in part, likely reflect changing laboratory practice.^13^

### Staphylococci

#### Staphylococcus aureus

**Figure 1.**
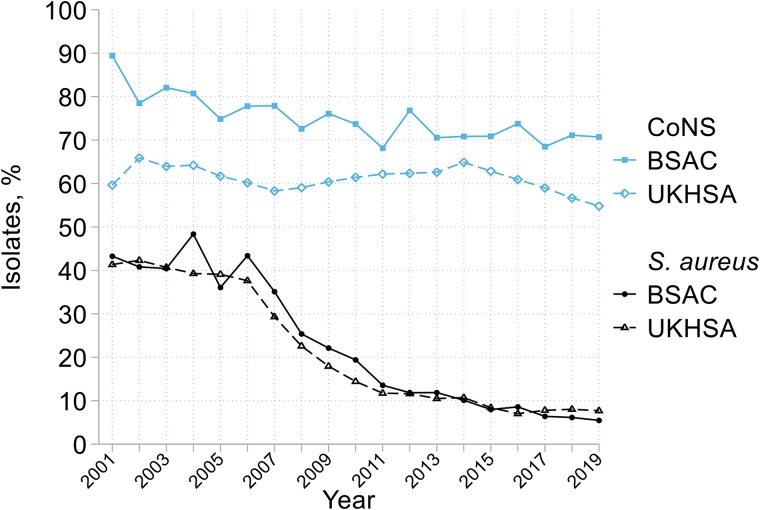
Methicillin resistance trends in *S. aureus* and CoNS from BSAC and UKHSA bacteraemia surveillance.

Both data sets (Figure 1) show major falls in the proportion of *S. aureus* bacteraemias due to MRSA, from *c.* 40% in 2001–06 to <10% by 2015–19. This decline largely occurred from 2006 to 2011; preceding and subsequent falls were slower, but continuous. *mecA* was sought from 2005 and confirmed in 1000/1022 (97.8%) of BSAC isolates phenotypically resistant to oxacillin (MIC >2 mg/L); the remaining 22 counted as likely BORSA (borderline oxacillin-resistant *S. aureus*), with oxacillin MICs of 4 mg/L (*N* = 19) or 8 mg/L (*N* = 3), though *mecC* was not excluded in all cases. These were from 18 centres and collected over 8 years, without time trend.

**Figure 2.**
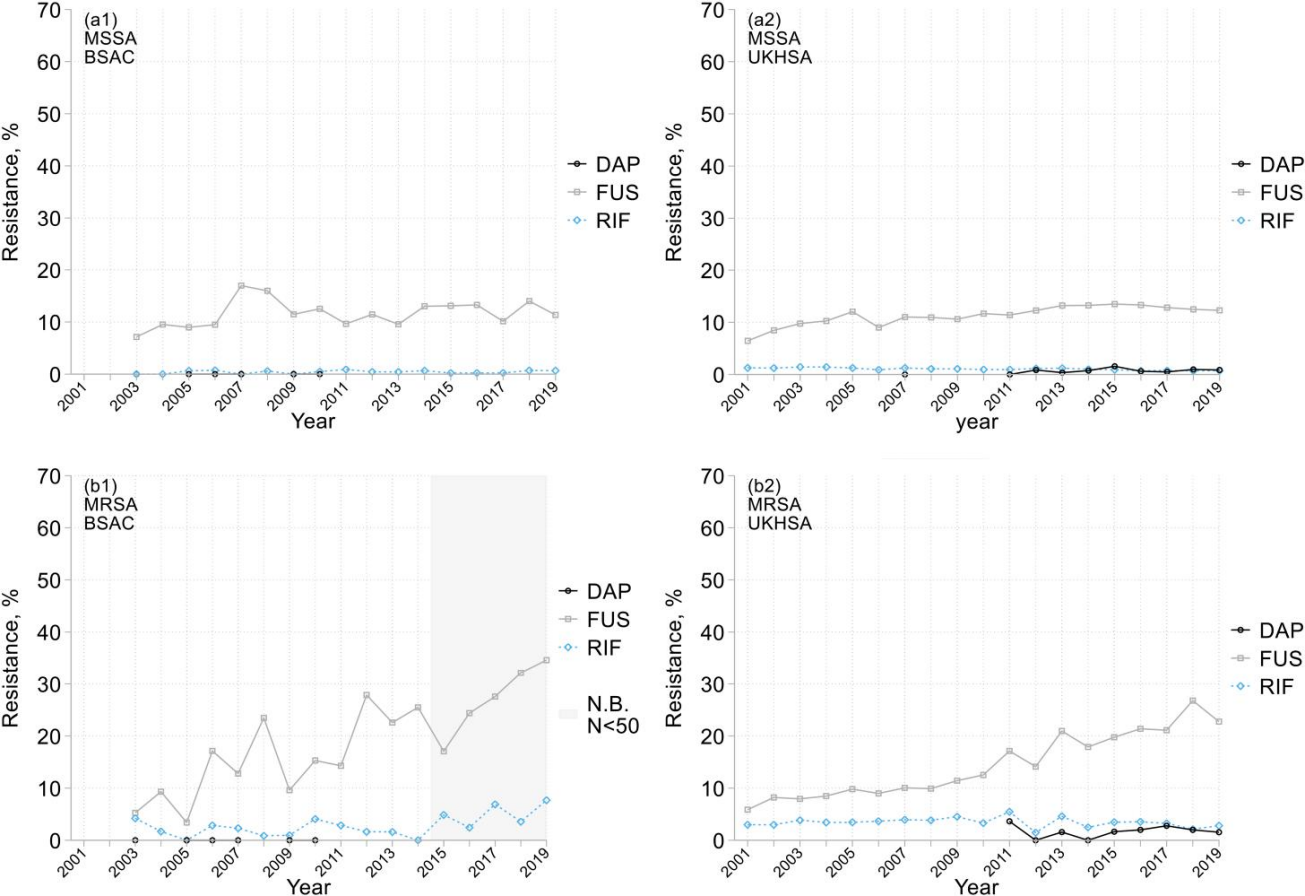
Resistance trends among (a) MSSA and (b) MRSA from bacteraemia in (1) BSAC and (2) UKHSA surveillance. DAP, daptomycin; FUS, fusidic acid; RIF, rifampicin. Not shown: linezolid, vancomycin, teicoplanin (<1% resistant overall in all panels, except teicoplanin 1.3% resistant overall in BSAC MRSA). Grey shading warns of very few BSAC MRSA isolates collected in 2015–19 (2641 per year). UKHSA data are not shown if <50 isolates tested; BSAC data for daptomycin in 2019 were excluded (testing discrepancy, see Supplementary Information).

Resistance trends for other antimicrobials included in the UKHSA extract are shown alongside BSAC results in Figure 2. Both datasets confirm that resistance remained extremely rare for oxazolidinones and vancomycin, whereas 1.3% of MRSA were resistant to teicoplanin. Resistance remained <5% for rifampicin in most years. Daptomycin was analysed by the BSAC from 2005 to 2010, with no resistance seen; rates up to 4% among UKHSA MRSA reports likely reflect laboratory issues for this difficult-to-test agent.^14^

There was a striking continuous increase in resistance to fusidic acid among MRSA, seen in both datasets, from *c.* 5% in 2001 to ≥25% by 2019, with a smaller increase among MSSA (Figure 2). Most of this resistance was low-level (MICs 2–16 mg/L), implying acquired *fusB*/*fusC*^15^; nevertheless, the relative increase over time was greater for high-level resistance, typically associated with chromosomal *fusA* mutation (5.2-fold versus 2.1-fold, comparing 2003–06 versus 2015–19 for MRSA).

BSAC data indicated major trends for antimicrobials not included in the UKHSA extract. Thus, among MRSA: (i) the prevalence of erythromycin resistance declined steadily from >80% to *c.* 60%, and (ii) ciprofloxacin resistance fell from *c.* 95% in the first decade to 84% by 2015–19. Resistance rates to erythromycin and ciprofloxacin were lower among MSSA, at 14% and 9%, respectively, for all years pooled, without clear trend. Clindamycin resistance, reviewed over 2012–19, tracked erythromycin resistance, at 60% for MRSA and 13% for MSSA; four-fifths was inducible.

The prevalence of tetracycline resistance rose from *c.* 3% to *c.* 10% among the diminishing annual numbers of MRSA collected, whilst trimethoprim resistance rose from <20% to *c.* 30%, reviewed against the *R* > 4 mg/L urinary breakpoint. The prevalence of both these resistances remained <5% among MSSA. Gentamicin MICs >2 mg/L, implying an acquired gene, were seen for 8% of MRSA but only 1% of MSSA overall. Mupirocin was tested from 2006. Low-level resistance (MICs, 2–64 mg/L) was much more prevalent in MRSA than MSSA (7.1% versus 0.2%) but half of the MICs signifying resistance were borderline at 2 mg/L; *mupA*-positive isolates comprised only 3.1% of MRSA and 0.4% of MSSA; 90% had high-level resistance (MIC >64, often ≥2048, mg/L).

Resistance rates in the BSAC surveillance, for all years pooled, remained <1% for both MRSA and MSSA for ceftobiprole, ceftaroline, tedizolid and tigecycline—which were not sought in the UKHSA extract as they are not widely tested—as well as for vancomycin, daptomycin and linezolid. Except for the cephalosporins, MIC distributions (see Appendix in Supplementary Data) for all these agents were narrow and similar for MSSA and MRSA, with >99% of MICs within ±1 dilution of the mode. Modes and (ranges) were: daptomycin 0.5 (0.12–1) mg/L; linezolid 2 (≤0.5–8) mg/L; tedizolid 0.25 (0.12–0.5) mg/L, with the drug not tested in years when linezolid-resistant isolates were recorded; tigecycline 0.25 (≤0.03–2) mg/L and vancomycin 1 (≤0.5–4) mg/L. MICs for ceftobiprole and ceftaroline were higher for MRSA (modes of 2 and 1 mg/L respectively, versus 0.5 and 0.25 mg/L for MSSA), with fuller comparisons published previously.^16^ Occasional resistance (<2% among both MRSA and MSSA) was seen for minocycline (mode MIC 0.12 mg/L; range 0.015 to ≥4) mg/L; more than for tigecycline but much less than for tetracycline.

The proportion of *S. aureus* with vancomycin MICs of 2 mg/L (i.e. on the upper edge of the susceptible range) was 28% for MSSA and 21% for MRSA, based on all years pooled. This proportion exceeds that in the global SENTRY surveillance, which consistently finds MIC_90_s of 1 mg/L,^17^ and may reflect the use of agar dilution in the BSAC Programme.

#### Coagulase-negative staphylococci

CoNS collected in the BSAC surveillance were identified by PCR from 2001 to 2005 and by MALDI-TOF from 2013 to 2019; collected CoNS were not identified to species level between 2006 and 2012. *Staphylococcus epidermidis* comprised most (65%) isolates, then *Staphylococcus hominis* (13%), *Staphylococcus haemolyticus* (11%) and *S. capitis* (7%). Proportions changed little over time, but the fraction identified only as ‘CoNS’ fell from 7% in the ‘PCR period’ to <1% with MALDI-TOF. CoNS reported to the UKHSA are increasingly identified to species level, showing broadly similar patterns though with, by 2019, *S. epidermidis* and *S. haemolyticus* accounting for only 59.5% of identified isolates versus 75% in the BSAC series.^13^ The BSAC, requesting ‘clinically significant CoNS’, possibly represents a somewhat different population to the UKHSA, accepting all blood culture reports.

**Figure 3.**
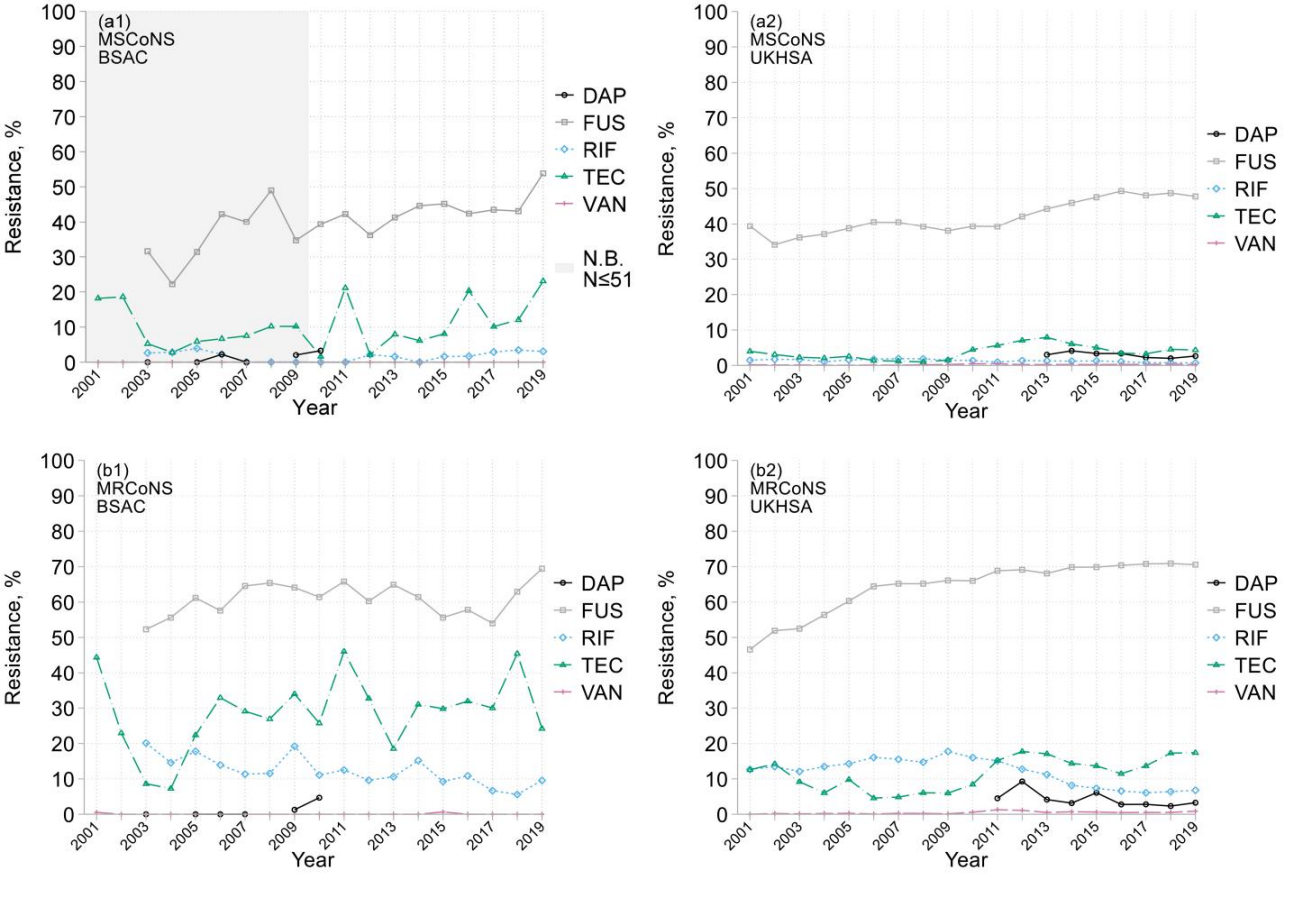
Resistance trends among (a) MSCoNS and (b) MRCoNS from bacteraemia in (1) BSAC and (2) UKHSA surveillance. DAP, daptomycin; FUS, fusidic acid; RIF, rifampicin; TEC, teicoplanin; VAN, vancomycin. Not shown: linezolid (<1% resistant overall in all panels). Shading warns of very few BSAC MSCoNS isolates were collected in 2001–09 (22–51 per year). UKHSA data are not shown if <50 isolates tested; BSAC data for daptomycin in 2019 were excluded (testing discrepancy, see Supplementary Information).

BSAC surveillance showed methicillin resistance falling slightly, from *c.* 80% in 2002–07 to *c.* 70% in 2013–19 (Table 1 and Figure 1); lower rates, but a similar trend, were recorded by the UKHSA (Figure 1). For other agents UKHSA data showed trends more clearly than BSAC data, which were compatible, but had greater statistical noise (i.e. random year-to year-fluctuation) reflecting a smaller sample size (Table 1 and Figure 3). Resistance to fusidic acid increased markedly, from *c.* 50% to 70% among methicillin-resistant (MR) CoNS and from 40% to 50% among methicillin-susceptible (MS) CoNS. Most of this resistance was low level, again implying *fusB/C*^15^; high-level resistance (MIC >32 mg/L) remained <3%, even among MRCoNS. Rifampicin resistance remained <2% among MSCoNS; among MRCoNS it fell from 12%–18% in 2001–10 to 6%–8% in 2014–19. Teicoplanin resistance fluctuated without clear trend, reaching 10%–20% in MRCoNS and 5%–10% among MSCoNS in 2010–19 based on UKHSA data, with higher rates in BSAC surveillance, perhaps reflecting the different species mix.

**Table 1.** Staphylococci: prevalence of resistance by species and period (BSAC surveillance)

Drug/mechanism	*S. aureus*					Coagulase-negative staphylococci								
	Breakpoint R > (mg/L)	% Resistant/positive				Breakpoint R > (mg/L)	% Resistant/positive							
		MSSA		MRSA			*S. epidermidis*		*S. hominis*		*S. haemolyticus*		*S. capitis*	
		2001–05	2013–19	2001–05	2013–19		2001–05	2013–19	2001–05	2013–19	2001–05	2013–19	2001–05	2013–19
N^a^		702	3124	504	278		615	981	107	205	123	149	72	106
*mecA*/oxacillin^b^	(2)	0	0	100	100	(0.25)	83.3	74.9	74.8	62.0	90.2	91.9	68.1	49.1
*mupA* ^c^	N/A	x	0.4	x	2.2	N/A	x	23.6	x	12.2	x	11.4	x	3.8
Ceftobiprole^c,d^	2	0*	0	1.0*	0	(2)^d^	6.4*	1.0	25.4*	6.3	34.8*	67.8	2.0*	0.9
Ciprofloxacin	1	9.1	8.5	96.8	85.3	1	56.4	61.3	33.6	28.3	74.0	79.9	29.2	15.1
Clindamycin^e^ constitutive	0.25	1.0	1.9	21.0	9.0	0.25	21.6	34.8	17.8	12.2	15.4	21.5	13.9	3.8
Clindamycin^c,e^ total	(0.5)	x	13.5	x	56.5	(0.5)	x	54.9	x	48.8	x	57.7	x	23.6
Daptomycin^c^	1	0*	x	0*	x	1	0*	x	x	x	x	x	x	x
Erythromycin	2	14.7	15.2	81.5	61.2	2	69.1	71.6	66.4	69.8	82.1	95.3	48.6	29.2
Fusidic acid^c^	1	8.5*	12.1	6.3*	25.2	1	51.1*	61.1	70.1*	76.6	50.0	28.2	x	35.8
Gentamicin^f^	(2)^f^	0.3	1.0	9.7	10.1	(2)^f^	57.2	57.4	41.1	28.3	74.0	88.6	51.4	43.4
Linezolid^c^	4	0	0*	0	0*	4	0	0.3*	0	0*	0	x	0	x
Minocycline^c^	0.5	0.5*	1.1*	1.2*	2.6*	0.5	3.8*	8.3*	2.2*	2.4*	18.0*	10.9*	x	0*
Rifampicin^c^	0.06	0.2*	0.4	2.0*	3.2	0.06	14.6*	10.2	7.5*	0.5	16.7*	6.7	x	0.9
Tedizolid^c^	0.5	x	0*	x	0*	0.5	x	0.1*	x	0*	x	0*	x	0*
Teicoplanin	2	0.6	0.4	1.4	0.7	4	22.0	32.6	3.7	9.3	31.7	12.8	9.7	17.0
Tetracycline	2	3.6	4.0	2.8	10.1	2	21.5	41.0	38.3	31.2	35.8	26.2	16.7	5.7
Tigecycline^c^	0.5	0*	0.2*	0*	0*	0.5	1.6*	8.3*	x	x	x	x	x	x
Trimethoprim^g^	4	2.6	5.0	17.1	27.0	4	65.9	64.8	71.0	62.9	65.9	77.2	27.8	17.9
Vancomycin	2	0	0	0.2	0.4	4	0	0	0	0	0.8	0	0	0.9

^a^ *N* of isolates if tested in all years of period. Caution: small numbers in some combinations, especially for CoNS species other than *epidermidis* and for antimicrobials tested intermittently.

^b^Methicillin-resistant: *mecA*-positive or, in 2001–04 only (prior to *mecA* testing), resistant to oxacillin at breakpoint shown.

^c^Not tested, or data not included, in every season of 2001–05 and 2013–19; periods of incomplete results shown by *. See Supplementary Information for detail.

^d^No EUCAST breakpoint for CoNS/ceftobiprole; results for CoNS are categorized using *S. aureus* breakpoints.

^e^Clindamycin resistance: constitutive = MIC >0.25 mg/L tested alone; total (2012–19 only) = constitutively resistant plus MIC >0.5 mg/L in the presence of 4 mg/L erythromycin.

^f^EUCAST ‘Breakpoint in brackets’ to detect acquired resistance mechanisms likely to undermine gentamicin’s synergistic contribution to the effectiveness of combination therapy.

^g^Clinical breakpoint for uncomplicated UTI only. Trimethoprim is of limited relevance outside UTI, but co-trimoxazole is occasionally advocated for wider use in the NHS.

x = Not tested, no breakpoint, or estimate based on <50 isolates not shown; 0 = No resistant isolates detected.

Data excluded (methodological issues identified in MIC distributions): daptomycin 2019; tigecycline 2002–03.

CoNS species were identified by PCR of DNA encoding 16S ribosomal RNA in 2001–05 and by MALDI-TOF from 2013 to 2019. Species were not identified 2006–12.

Resistance to the oxazolidinones and vancomycin remained <1% in both datasets. Just 2/3954 BSAC isolates were resistant to vancomycin, both with MICs of 8 mg/L versus the 4 mg/L breakpoint; MICs of 4 mg/L were recorded for a further 311 (7.9%). Daptomycin resistance averaged 3% for MSCoNS and 4% for MRCoNS, based on UKHSA data from 2011 to 2019, with the same caveats as for *S. aureus*. BSAC data were too variable to convincingly describe resistance trends for antimicrobials not also included in the UKHSA extract. Nevertheless, resistance to several drugs was highly prevalent among MRCoNS, with 60%–80% all-years rates for ciprofloxacin, clindamycin (nearly half inducible), erythromycin, gentamicin and trimethoprim, and 36% for tetracycline, though only 8% for minocycline. Across 2007–19, 5% of MSCoNS and 26% of MRCoNS had *mupA*, almost all with high-level mupirocin resistance (MIC >64 mg/L), whereas only <2% and 5% of MSCoNS and MRCoNS, respectively, had low-level *mupA*-independent resistance (MICs 2–64 mg/L). A resistance rate of 13% was found for ceftobiprole (using the *S. aureus* breakpoint) almost entirely reflecting MR-*S. haemolyticus* with MICs of 4 mg/L,^16^ and a 6% rate for tigecycline, mostly with MICs of 1 mg/L.

MIC distributions for most broadly-active agents (see Appendix to Supplementary Data) were little wider than for *S. aureus*; however, distributions for ceftaroline and ceftobiprole were significantly wider, reflecting higher values for MRCoNS, and, especially, MR *S. haemolyticus.*^16^ MIC modes and ranges were: ceftaroline (mode 0.25; range ≤0.002 to 4 mg/L); ceftobiprole (1; 0.008 to 8 mg/L); daptomycin (0.5;  ≤ 0.03 to 2 mg/L); linezolid (1;  ≤ 0.5 to >8 mg/L); tedizolid (0.25; 0.12 to 2 mg/L, with the highest value for an isolate with a linezolid MIC >8 mg/L); vancomycin (2;  ≤ 0.5 to 8 mg/L).

The proportion of methicillin resistance fell over time for all species except *S. haemolyticus* (Table 1) where it remained *c.* 90%–92%. Resistance to fusidic acid and teicoplanin declined among *S. haemolyticus* but increased in other species; rifampicin resistance declined universally.

### Enterococci

Some 4486 enterococci were collected by the BSAC surveillance, principally *E. faecalis* (2428, 54%) and *E. faecium* (1824, 41%), together with 75 *E. durans*, 33 *E. gallinarum*, 30 *E. avium*, 24 *E. casseliflavus*, 18 *E. raffinosus*, 2 *E. hirae* and 52 identified only to genus level (Supplementary Data, Tables S4b; S7a and b). The proportion of *E. faecium* increased from 31% in 2001–03 to 51% in 2017–19, at the expense of *E. faecalis.*^8^ This trend (Table S4b) was corroborated by UKHSA data, showing increases over time in resistances to ampicillin and vancomycin, which are wholly or largely associated with *E. faecium*. We noted a fall in high-level resistance to gentamicin (MIC >128 mg/L) and ciprofloxacin (MIC > 16 mg/L) among *E. faecalis* but no trend in either major species for: ampicillin (overall, 0/96% resistance among *E. faecalis*/*E. faecium*, respectively), linezolid (<1/<1%), teicoplanin (2/28%), tigecycline (<1/<1%) or vancomycin (2/29%) (Table 2). Over 95% of vancomycin-resistant *E. faecium* (502/530) and *E. faecalis* (52/54) were cross-resistant to teicoplanin, implying dominance of VanA, though direct molecular investigation was not undertaken. Vancomycin resistance at 8 mg/L was frequent in *E. gallinarum* (25/33) and *E. casseliflavus* (8/24), which have endogenous *vanC*^18^; ‘borderline’ vancomycin MICs of 4 mg/L were frequent for ‘susceptible’ isolates of these species, whereas full teicoplanin susceptibility consistently was retained (Table S7b).

**Table 2.** Enterococci: prevalence of resistance by species and period (BSAC surveillance)

Antimicrobial	Breakpoint *R* > (mg/L)	% Resistant							
		*E. faecalis*				*E. faecium*			
		2001–04	2005–09	2010–14	2015–19	2001–04	2005–09	2010–14	2015–19
*N* ^a^		595	645	637	551	292	409	538	585
Ampicillin	8	0	0.2	0	0	88.0	95.6	99.6	95.7
Gentamicin^b^	128	51.4	46.7	33.9	21.4	44.2	43.0	55.0	49.2
Linezolid^c^*	4	0	0.2	0*	0*	0	0.2	0.5*	0.8*
Teicoplanin	2	3.4	2.9	1.3	1.5	19.9	28.1	30.1	28.7
Tigecycline^c^*	0.25	0*	1.1	0*	x	0*	0.2	0.9*	x
Vancomycin	4	3.2	2.6	1.6	1.5	21.6	31.1	31.6	29.1

^a^ *N* of isolates if tested in all years of period.

^b^Screening breakpoint for high-level aminoglycoside resistance likely to undermine synergy in combination therapy.

^c^Not tested, or data not included, in every season; periods of incomplete results shown by *. See Supplementary Information for detail.

x = Not tested, no breakpoint, or estimate based on <50 isolates not shown; 0 = No resistant isolates detected.

Data excluded (methodological issues identified in MIC distributions): tigecycline 2002–03.

Oxazolidinones and tigecycline were active against >99% of enterococci, with >99% of MICs clustered at 1–2 mg/L for linezolid and 0.25–0.5 mg/L for tedizolid (see MIC distribution in Appendix to the Supplementary Data). Just nine enterococci (0.2%), scattered across 2005–19, were resistant to linezolid (MICs >4 mg/L) or were not inhibited by tedizolid (which lacks EUCAST breakpoint for the genus) at 0.5 mg/L—seven *E. faecium*, one *E. faecalis* and one unidentified enterococcus with linezolid MICs ≥8 mg/L. Tigecycline MICs ranged from ≤0.03 to 2 mg/L and mostly were higher for *E. faecalis* (mode 0.12 mg/L) than for *E. faecium* and other/unidentified enterococci (modes 0.06 mg/L). EUCAST^19^ does not define a daptomycin breakpoint or ECOFF for enterococci, but the drug is of interest in enterococcal endocarditis owing to a lack of bactericidal alternatives.^20^ MICs, determined up to 2010, were slightly lower for *E. faecalis* (91% of values 0.5–1 mg/L) than *E. faecium* (91% at 1–2 mg/L), with values of 4 mg/L for <1% of *E. faecalis* (3/809), 6% of *E. faecium* (32/518) and 7% of other/unidentified enterococci (6/58). No daptomycin MICs of 8 mg/L were seen, but the MIC for one isolate lacking species identification was ≥16 mg/L.

### Streptococcus pneumoniae

**Figure 4.**
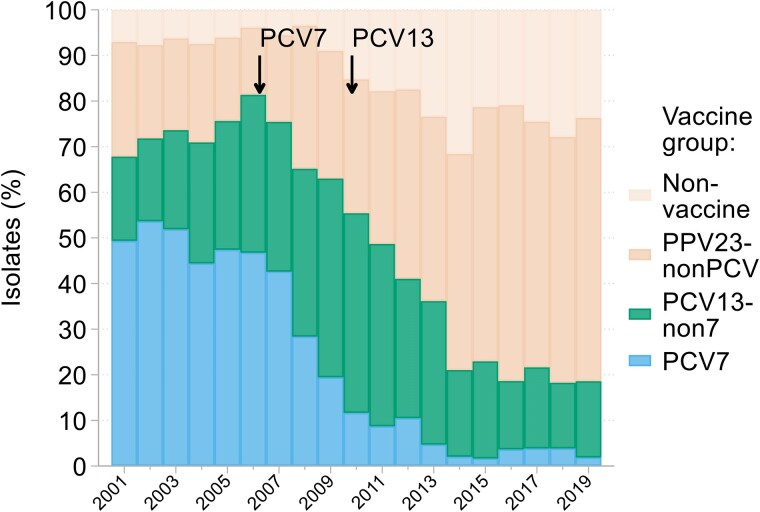
Changing prevalence of vaccine serotypes among *S. pneumoniae* from BSAC bacteraemia surveillance. Arrows indicate when PCV7 and PCV13 were first introduced to the infant vaccination schedule in England. Serotypes included in each vaccine group were: PCV7–4, 6B, 9V, 14, 18C, 19F, 23F; PCV13-non7–1, 3, 5, 6A, 7F, 19A; PPV23-nonPCV—2, 8, 9N, 10A, 11A, 12F, 15B, 17F, 20, 22F, 33F; non-vaccine—any serotype not included in PCV7, PCV13 or PPV23.

All 4301 *S. pneumoniae* isolates collected in the BSAC surveillance were serotyped. Proportions within the coverage of different vaccines changed substantially over time (Figure 4); details are discussed elsewhere.^21^

**Figure 5.**
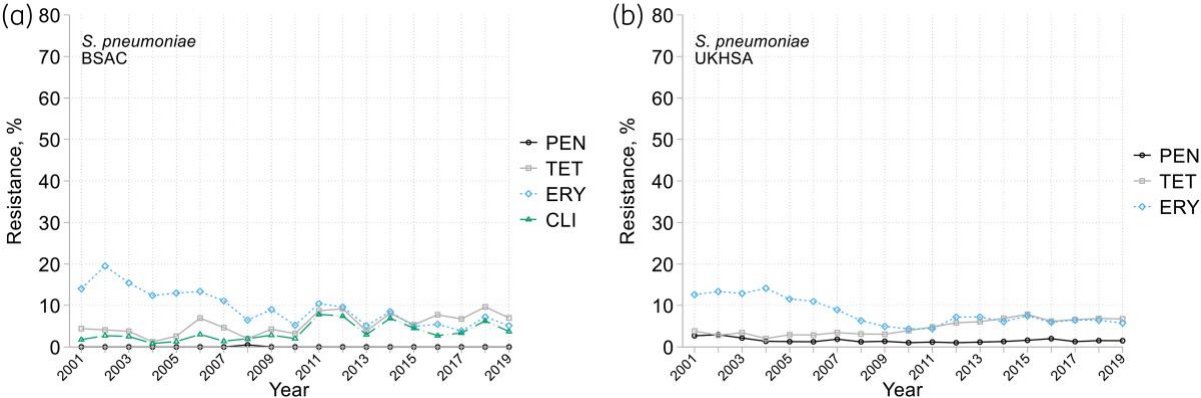
Resistance trends among *S. pneumoniae* from bacteraemia in (a) BSAC and (b) UKHSA surveillance. PEN, penicillin; TET, tetracycline; ERY, erythromycin; CLI, clindamycin. Clindamycin not shown in (b) as not included in UKHSA data extract.

Both surveillance systems showed substantial falls in erythromycin resistance in the first decade, from *c.* 12%–15% to *c*. 5%–8% along with smaller, but unequivocal, *increases* in tetracycline resistance, from 3%–4% to *c.* 7% during the second decade (Figure 5). BSAC surveillance detected just one isolate truly ‘resistant’ to penicillin—a serotype 19F organism from 2008, MIC 4 mg/L; however, 5.9% of isolates counted as ‘susceptible, increased exposure’ (MIC 0.12–2 mg/L), without time trend. UKHSA data recorded 1%–2% resistance to penicillin throughout, with many ‘resistant’ isolates likely categorized against EUCAST’s 0.12 mg/L screening breakpoint.

BSAC surveillance included 10 antimicrobials not included in the UKHSA extract (Figure 5). Clindamycin resistance increased from an average of 2% in 2001–04 to 4% in 2015–19, whereas erythromycin resistance declined from 15% to 5% over the same period. Resistance rates for other β-lactams were similarly low as for penicillin, at <1% for amoxicillin and ceftobiprole, with no resistance seen for cefotaxime, ceftaroline or carbapenems nor for glycopeptides or linezolid (see MIC distributions in Appendix to Supplementary Data). MIC modes and ranges were: amoxicillin (0.015;  ≤ 0.004–8 mg/L); ceftobiprole (0.015;  ≤ 0.004–1 mg/L); cefotaxime (0.015;  ≤ 0.004–2); ceftaroline (0.008;  ≤ 0.004–0.25 mg/L); imipenem (0.004;  ≤ 0.002–0.5 mg/L); meropenem (0.008;  ≤ 0.002–1 mg/L); teicoplanin (0.06;  ≤ 0.03–0.5 mg/L); vancomycin (0.5; 0.12–2 mg/L); linezolid (1; 0.5–2 mg/L).

### Other α- and non-haemolytic streptococci

The BSAC collection included 3494 α- and non-haemolytic streptococci besides pneumococci. These divided as: 58% *S. mitis*, 19% *S. anginosus*, 12% *S. bovis*, 9% *S. salivarius* and 2% *S. mutans* groups (Table 3). There was no clear trend in species distribution over time (Table S4b).

**Table 3.** Non-pneumococcal α- and non-haemolytic streptococci: prevalence of resistance by group (BSAC surveillance, based upon all years combined)

Drug/mechanism	Breakpoint *R* > (mg/L)	% Resistant					
		*S. mitis group*	*S. anginosus* group	*S. bovis* group	*S. salivarius* group	*S. mutans* group	Total^a^
*N* ^a^ (%)		2032 (58.2)	664 (19.0)	410 (11.7)	318 (9.1)	68 (1.9)	3492 (100)
Cefotaxime	0.5	6.9	0.5	0.5	3.5	1.5	4.5
Clindamycin^b^ constitutive	0.5	7.8	4.7	12.4	7.5	1.5	7.6
Clindamycin^b,c^ total	(0.5)	9.5	4.9	31.5	8.2	2.8	10.8
Erythromycin^c^†	0.25	[51.4]†	[10.7]†	[24.8]†	[43.2]†	[1.6]†	[38.7]†
Gentamicin^d^	128	<0.1	0	0.2	0	0	<0.1
Penicillin	2	2.5	0	0	1.3	0	1.5
Penicillin ‘I’^e^	[0.25–2]^e^	15.2	0.8	0.7	19.5	1.5	10.9
Tedizolid^c^	0.5	x	0	x	x	x	X
Teicoplanin	2	0	0	0	0	0	0
Tetracycline	2	28.6	19.3	69.3	16.4	1.5	30.0
Vancomycin	2	0	0	0	0	0	0

^a^ *N* of isolates if tested in all years of period. Total excludes two isolates of unidentified groups.

^b^Clindamycin resistance: constitutive = MIC >0.5 mg/L tested alone; total (based upon 2012–19 only) = constitutively resistant plus isolates with MIC >0.5 mg/L in the presence of 4 mg/L erythromycin.

^c^Not tested, or data not included, in every season. See Supplementary Information for detail.

^d^Screening breakpoint for high-level aminoglycoside resistance likely to undermine synergy with other active therapy.

^e^Penicillin ‘susceptible, increased exposure’ i.e. 0.25 < MIC ≤ 2 mg/L.

[]† N.B. overall prevalence of resistance changed substantially over time (see text): the figures given, averaged over time, serve only to highlight differences between species groups.

x = No applicable breakpoint. 0 = no resistant isolates detected.

Data excluded (methodological issues identified in MIC distributions): erythromycin 2003–04.

**Figure 6.**
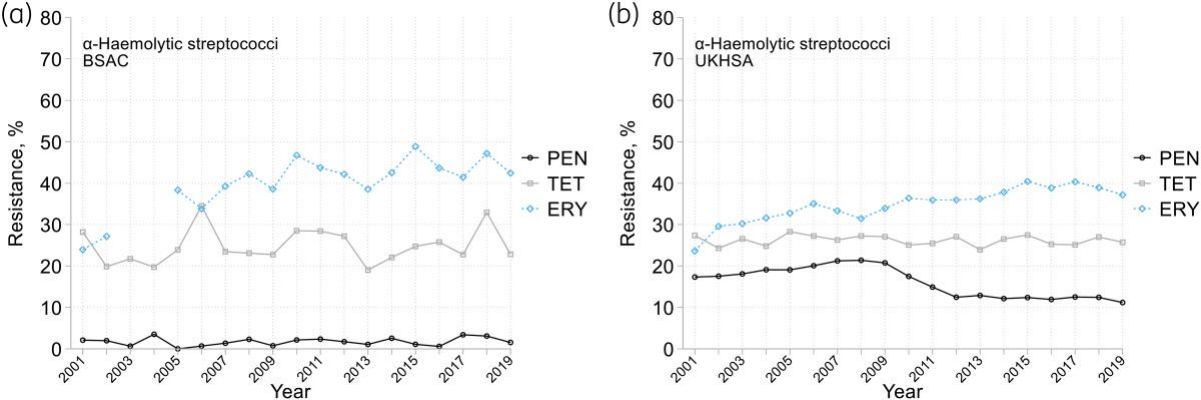
Resistance trends among non-pneumococcal α-haemolytic streptococci from bacteraemia in (a) BSAC and (b) UKHSA surveillance. PEN, penicillin; TET, tetracycline; ERY, erythromycin. BSAC surveillance included *S. bovis* group together with α-haemolytic streptococci for collection and analysis, but they are excluded here, as (i) non-haemolytic and (ii) to match the UKHSA data extract. BSAC erythromycin data were excluded for 2003–04 (testing discrepancy, see Supplementary Information).

The UKHSA extract (which included only α-haemolytic species, not *S. bovis* nor non-haemolytic groups) showed erythromycin resistance gradually increasing, from *c.* 30% to *c.* 40%, whereas tetracycline resistance was stable at 25%–30%; BSAC data were comparable, but with greater year-to-year variation (Figure 6). Penicillin resistance (MIC >2 mg/L) averaged <2% in BSAC data, but with 12% of isolates counting as ‘susceptible, increased exposure’ (MICs 0.5–2 mg/L). The UKHSA recorded higher resistance rates, with a distinct drop from *c.* 20% before 2009 to *c*. 10% from 2012. Adjustments to BSAC and EUCAST breakpoints in 2010 and 2012 may explain this fall, whilst the generally higher UKHSA rate probably reflects inclusion of isolates scored as ‘Susceptible, increased exposure’ by the BSAC. BSAC surveillance recorded no isolates resistant to vancomycin or teicoplanin and <1% high-level resistance to gentamicin.

Resistance patterns differed among groups (Table 3). Averaged over all 19 years, erythromycin resistance was most prevalent in the *mitis* group (increasing from 32% in 2001–04 to 57% in 2015–19), ahead of the *salivarius* (32% rising to 47%), *bovis* (20% rising to 31%) and *anginosus* (stable around 11%) groups. Clindamycin resistance (constitutive plus inducible, tested 2012–19) was <10% in all groups except *S. bovis* (31%, approximately half inducible). Tetracycline resistance was most prevalent in the *bovis* group (69%), followed by the *mitis* (29%), *anginosus* (19%) and *salivarius* (16%) groups. Cefotaxime resistance—recorded for 7% and 4% of the *mitis* and *salivarius* groups, respectively, but <2% among others—was borderline (MIC, 1 mg/L) in over half of cases. *S. mutans* group isolates were particularly susceptible, with resistance <2% for all tested antimicrobials with breakpoints (see MIC distributions in Appendix to Supplementary Data).

Five agents tested in BSAC surveillance lacked breakpoints for some or all groups. Ceftobiprole MICs ranged from ≤0.004 to ≥16 mg/L, with modes of 0.015 or 0.03 mg/L for all groups except *S. anginosus* (0.12 mg/L); MICs ≥0.5 mg/L were concentrated in the *S. mitis* group (10%, versus <2% for other groups, with 9/10 values ≥4 mg/L being for the *S. mitis* group). Daptomycin MICs ranged from ≤0.03 to 4 mg/L, with higher modes for *S. mitis* and *S. anginosus* (0.5 mg/L) than for the *S. bovis* and *S. salivarius* groups (0.12 and 0.06 mg/L, respectively). Linezolid had narrow MIC distributions, with modes at 2 mg/L (*S. bovis* group) and 1 mg/L (all others) and a range from ≤0.25 to 2 mg/L. Tedizolid had similarly narrow ranges (0.12–0.5 mg/L), with 95% of MICs at the 0.25 mg/L mode. Tigecycline MICs ranged from ≤0.015 mg/L to 1 mg/L; its mode was higher (0.12 mg/L) for *S. bovis* and *S. mutans* than for other groups (0.06 mg/L) (see MIC distributions in Appendix to Supplementary Data). No convincing time trends were seen.

### β-Haemolytic streptococci

A total of 4372 β-haemolytic streptococci were collected: 1560 group A, 1500 group B, 1007 group G, 299 group C isolates and six undifferentiated group C/G. The species composition shifted progressively from 2006, with group B declining from *c.* 40% to *c.* 30% by 2017–19, while group C/G increased from 30% to 40%. This change was more obvious in the larger UKHSA dataset. A spike of group A streptococci in 2003–04, up to *c.* 40% from its usual 30%, was apparent in both datasets.

**Figure 7.**
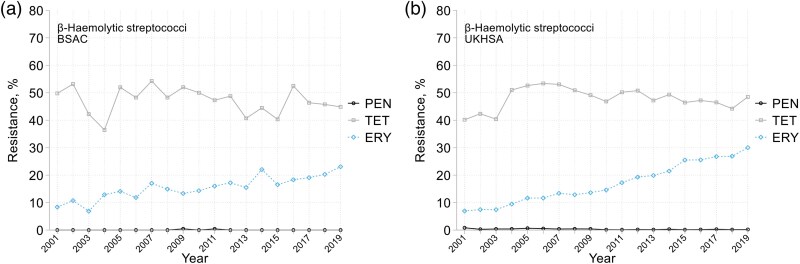
Resistance trends among β-haemolytic streptococci from bacteraemia in (a) BSAC and (b) UKHSA surveillance. PEN, penicillin; TET, tetracycline; ERY, erythromycin.

The overall prevalence of erythromycin resistance rose steadily from *c.* 10% in both surveillances in 2001 to between 20% (BSAC) and 30% (UKHSA) by 2019 (Figure 7), doubling among groups B and C/G isolates though remaining stable at *c.* 4% in group A (Table 4). There was little change in the prevalence of resistances to tetracycline (13%, 84% and 45% in groups A, B and C/G, respectively) or penicillin (universally <1%).

**Table 4.** β-Haemolytic streptococci: prevalence of resistance by group (BSAC surveillance)

Drug/mechanism	Breakpoint *R* > (mg/L)	% Resistant			
		Group A	Group B	Group C/G	Total
*N* ^a^		1560	1500	1312	4372
Clindamycin^b^ constitutive	0.5	[1.1]†	[11.6]†	[6.2]†	[6.2]†
Clindamycin^b,c^ total	(0.5)	[1.9]†	[20.2]†	[19.6]†	[13.9]†
Daptomycin^c^	1	0	0	0	0
Erythromycin	0.5	[4.0]†	[20.8]†	[23.1]†	[15.5]†
Linezolid^c^	2	1.1	0.4	1.4	0.9
Penicillin	0.25	0	0.1	0	<0.1
Tedizolid^c^	0.5	0	0	0	0
Teicoplanin	2	0	0.1	0	<0.1
Tetracycline	2	13.3	83.9	45.3	47.1
Tigecycline^c^	0.12	0.7	5.5	25.6	9.4
Vancomycin	2	0	0	0	0

^a^ *N* of isolates if tested in all years of period.

^b^Clindamycin resistance: constitutive = MIC >0.5 mg/L tested alone; total (2012–19 only) = constitutively resistant plus isolates with MIC >0.5 mg/L in the presence of 4 mg/L erythromycin.

^c^Not tested, or data not included, in every season. See Supplementary Information for detail.

[]† N.B. overall prevalence of resistance changed substantially over time (see text): the figures given, averaged over time, serve only to highlight differences among species groups.

0 = no resistant isolates detected.

Data excluded (methodological issues identified in MIC distributions): tigecycline 2002–03.

In the BSAC surveillance, the prevalence of clindamycin resistance (constitutive plus inducible, with inducibility tested from 2012 to 2019) was 2% for group A and 20% for groups B and C/G isolates. Half this resistance was inducible in group C/G isolates, versus one-sixth in group B. Tigecycline overcame most tetracycline resistance in groups A and B; low-level tigecycline resistance (MICs, 0.25–0.5 mg/L) was frequent in group C/G (Table 4) though contingent on EUCAST’s 2018 reduction of the resistance breakpoint from >0.5 to >0.12 mg/L. We saw no isolates resistant to vancomycin, daptomycin or tedizolid;  < 1% were resistant to teicoplanin or linezolid, and just two group B isolates were penicillin resistant (MICs, 0.5 and 2 mg/L).

Over 98% of cefotaxime MICs were ≤0.06 mg/L, fewer than 0.2% were ≥0.25 mg/L; similarly, over 99% of ceftobiprole MICs were ≤0.06 mg/L with only nine higher values (6 at 0.12; 2 at 0.25 and 1 at 0.5 mg/L) recorded (see MIC distributions in Appendix to Supplementary Data).

Gentamicin MICs spread broadly: modes for groups A, B and C/G were 4, 8 and 4 mg/L, respectively; notably, group B isolates also had a thicker tail of higher MICs. Three isolates with gentamicin MICs >128 mg/L were collected—two group B isolates, described previously, with AAC(6′)-APH(2″)^22^ and one group C/G organism, not studied further.

Mode penicillin MICs were higher for group B (0.03 mg/L) than other groups (≤0.008 mg/L). Daptomycin MICs (range ≤0.03 to 1 mg/L overall) also had a higher mode for group B (0.25 mg/L) than groups A and C/G (both 0.06 mg/L). MICs for vancomycin (range ≤0.06 to 2, mode 0.5 mg/L) and teicoplanin (≤0.03 to 4, mode 0.12 mg/L) were tightly clustered, with >99% and 97% of values, respectively, within ±1 dilution of their modes. MICs also were tightly clustered for linezolid (range 0.5–4 mg/L; 99% at 1–2 mg/L) and tedizolid (range 0.12–0.5 mg/L; 99% at 0.25 mg/L).

## Discussion

In reviewing this surveillance, covering the period 2001–19, three patterns emerge. First, major trends that have received extensive review, notably the decline of MRSA and PCV-driven shifts in *S. pneumoniae* serotype epidemiology. Secondly, strong trends that have been less remarked, notably rising fusidic acid resistance among staphylococci, the rise of *E. faecium* relative to *E. faecalis* and the rise of CoNS in the UKHSA surveillance. Last, many organism/antibiotic pairs where resistance rates were little changed, including some where rising resistance was predicted.

The decline of bloodstream MRSA, from early in the BSAC surveillance, is well known.^4^ It followed the introduction of mandatory reporting in 2001 with annual reduction targets imposed in England from 2004. Most NHS Trusts adopted a ‘bundle’ approach in response, including pre-admission screening, decolonization, reinforced handwashing, alcoholic hand-rubs, dedicated teams to insert intravenous lines, reduced cephalosporin and fluoroquinolone prescribing, along with isolation of colonized or infected patients.^23^ These actions are generally agreed to have underpinned the subsequent reductions, with caveats that: (i) one of the two major epidemic (E)MRSA lineages, EMRSA-16, was already declining by 2001^24^ and (ii) it is unclear which bundle components mattered most. The lack of concurrent reductions for MSSA bacteraemias is notable, as some bundle components—e.g. dedicated teams to insert intravenous lines—should have impacted all *S. aureus*, whereas others—e.g. pre-admission decolonization—specifically targeted MRSA. EARS-net data show widespread continental declines in MRSA from 2001 to 2019 (e.g.: France 33.4% to 11.6%; Germany 15.7% to 6.7% and Italy 41.0% to 34.3%)^25^; nevertheless, the UK is an extreme case: at the upper edge of the European range in 2001 and the lower edge by 2019.

Changes in the serotype distribution of invasive *S. pneumoniae* following deployment of pneumococcal conjugate vaccines (PCV7 and PCV13) in 2006 and 2010, respectively likewise are well known.^6,7,21^ PCV7 and 13 suppressed most targeted types, but this has been partially offset particularly in adults, who were protected by a herd effect, by the rise of non-vaccine types.^26^ Before 2006, a macrolide-resistant serotype 14 lineage with *mefA* (efflux) was widespread in the UK.^27^ This was displaced by PCV7.^7^ Subsequently, there was a rise^28^ and peaking^21^ in non-vaccine serotype 15A isolates resistant to macrolides and tetracyclines and with diminished penicillin susceptibility. Macrolide resistance in these latter isolates was determined by *erm*(B), encoding an rRNA methyltransferase and also compromising clindamycin whereas *mefA* solely compromises macrolides.^28^ Rises and falls in resistance among bloodstream *S. pneumoniae* across Europe from 2005 to 2019 were erratic.^25^ What is consistent is that the vaccination of children with conjugate vaccines reduced the incidence of invasive pneumococcal infection.^6,26^

Turning to less-remarked changes, the most striking are the rises in fusidic acid resistance among MRSA, MSSA and CoNS. For MRSA and MSSA, fusidic acid resistance began from low baselines in 2001 (both 6% in the UKHSA dataset), reaching 23% for MRSA and 12% for MSSA by 2019; for CoNS they began from a high baseline of 40%–50%, according to methicillin status, reaching 71% and 48% among MRCoNS and MSCoNS respectively, by 2019. From BSAC data, most resistance was low level, implying *fusB/C* efflux, which generally is plasmid mediated.^15^ The widespread *S. aureus* Clonal Complex 121, associated with impetigo and *fusB*,^29^ is not associated with invasive infections and is rarely methicillin resistant. Nevertheless, it may be a vector for gene or plasmid spread, as may skin CoNS with *fusB/C*,^30^ with all these types favoured by widespread community use of topical fusidic acid.

Shifts in resistance among the diminishing numbers of bloodstream MRSA were seen. Falls in macrolide and clindamycin resistance and rises in trimethoprim and tetracycline resistance may reflect (i) loss or gain of genes by the dominant EMRSA-15/ST22 lineage and/or (ii) the gradual penetration of new MRSA lineages with characteristic antibiograms.^31^

For bloodstream CoNS, the major shift was the 7.8-fold rise in reports to the UKHSA compared with a 2.4-fold rise for all species (Table S15 and Figure S4). This is believed to substantially reflect altered reporting practice, not a rise in incidence. In support of this view, we note that species proportions within the BSAC sample—submitted as ‘clinically significant’—remained stable over time, whereas a true shift should predominantly involve one species, changing these proportions. Nonetheless, it is striking that many laboratories are performing identification and susceptibility testing on a large proportion of CoNS from blood culture, implying that they view the isolates as significant.^13^ A lower proportion of *S. epidermidis* and *S. haemolyticus* in the UKHSA data may contribute to lower resistance rates to methicillin and teicoplanin—traits concentrated in these species (though less so for teicoplanin and *S. haemolyticus* after 2013). Several multi-resistant global *S. epidermidis* lineages have been recognised recently, including in the UK.^32^ However, these typically are rifampicin resistant whereas, among the BSAC collection this trait declined slowly (Figure 3) suggesting that they were not expanding.

There were two major shifts among enterococci, highlighted elsewhere. First, both the BSAC and UKHSA surveillances show that the proportion of *E. faecium* increased relative to *E. faecalis*, with similar changes recorded internationally.^8^ By 2016, *E. faecium* outnumbered *E. faecalis* among enterococci received by the BSAC. Secondly, within *E. faecalis*, there was a decline of high-level gentamicin resistance. Early in the surveillance period high-level gentamicin resistance was strongly associated with two widespread *E. faecalis* lineages (as defined by their by pulse-field gel electrophoresis profiles or by WGS); these also had unusually high-level ciprofloxacin resistance,^8,33,34^ and these may have been displaced. The decline of *E. faecalis* versus *E. faecium* may reflect: (i) the decline of these two clones, which augmented early *E. faecalis* totals; (ii) selection of *E. faecium*, owing to its greater antibiotic resistance, or (iii) expanding patient populations vulnerable to *E. faecium*. The stability of prevalent vancomycin resistance in *E. faecium* here—and especially in Ireland (EARS-net, 2005, 30.9%; 2019, 38.4%)—contrasts with rising rates in Eastern Europe (e.g. Poland 2005, 4.8%; 2019, 44%; Bulgaria 2005, 0%; 2019, 12.1%) and stably low rates in France (2005, 2.6%; 2019, 0.7%).^25^

Among β-haemolytic streptococci there was a notable decline in the proportion of Group B (*S. agalactiae*) relative to groups C/G. Given that 29% of the group B isolates were from children <1 year old, this fall may reflect improved screening and intrapartum prophylaxis. A spike in group A isolates in 2003–04 was captured by both surveillances, tallying with recognition that the incidence of invasive infection due to this species fluctuates over time, though reasons remain uncertain.^35^ Rises in macrolide resistance among groups B and C/G isolates are notable, and contrast with the stability of other resistances in these species. A caveat is that the BSAC’s collection strategy, of a fixed annual quota of isolates, may have biased against groups that were most prevalent later in the year.

Perhaps the most remarkable observation is how much remains unchanged over nearly 20 years. The discovery of vancomycin-intermediate and -resistant *S. aureus* around Year 2000 prompted fear that we would ‘lose’ antistaphylococcal glycopeptides.^36^ This has not happened. Later, it was feared that mupirocin resistance would proliferate as use for MRSA decolonization expanded^9^; *mupA* is widespread in CoNS, and spillage into nasal MRSA seemed predictable. Again, it did not happen. Next, whilst there have been many literature reports of Gram-positive cocci resistant to oxazolidinones, daptomycin, anti-MRSA cephalosporins and tigecycline,^10^ none of these traits has proliferated. A few oxazolidinone-resistant *S. aureus* (1/5149 tested) and enterococci (9/4236) were collected, but without accumulation. Daptomycin resistance was absent from S*. aureus* during the six years it was analysed by the BSAC and, although UKHSA data record low rates of resistance, these lacked trend and likely reflect testing issues. A 12% rate of ceftobiprole resistance among MRCoNS looks striking, but was almost entirely due to ‘borderline’ MICs of 4 mg/L for *S. haemolyticus.*^16^ The MIC distribution for this species is simply higher than for other staphylococci, without ongoing creep. Strikingly-prevalent tigecycline resistance among Group C/G streptococci (25.6%) reflects EUCAST’s 2018 breakpoint reduction, not bacterial evolution. Lastly, enterococcal vancomycin resistance, which fluctuated between 0.7% and 4% for *E. faecalis* and 19% and 40% for *E. faecium*, deserves consideration. Similar rates pertained during the 1990s,^37^ before the EU’s 1997 ban on avoparcin as a feed additive. Dramatic rises in vancomycin-resistant *E. faecium* in Eastern Europe long post-date that ban,^25^ and typing indicates that resistance is strongly associated with the human-adapted A1 clade.^38^ Persistence of this lineage appears to be the driver of resistance, not dissemination via the food chain.

These findings—from two complementary surveillances—present an encouraging picture. MRSA and resistant pneumococci have declined; the situation with enterococci has worsened only modestly, largely owing to a species shift; there was no substantial change in resistance among non-pneumococcal streptococci. Rising fusidic acid resistance among staphylococci is interesting, but the drug is little used in severe infections. The data belie apocalyptic assertions that we ‘face a return to the pre-antibiotic era’. In the pre-antibiotic era Gram-positive cocci caused *c.* 90% of bacteraemia; *S. pyogenes* alone accounted for 50%, with high mortality.^3^ There is no imminent danger of returning to this position given the many antibiotics with near-universal activity against Gram-positive species.

## Supplementary Material

dkaf249_Supplementary_Data

## Transparency declarations

S.M., R.H., A.C., R.A. and O.N. are employees of the UKHSA. R.H. and O.N. work for the Antimicrobial Resistance and Healthcare Associated Infections Division and have no conflict of interest to declare. S.M., A.C. and R.A. work for the Antimicrobial Resistance and Healthcare Associated Infections Reference Unit (AMRHAI), which has received financial support for conference attendance, research projects or contracted evaluations from numerous sources, including Accelerate Diagnostics, Allecra Therapeutics, AstraZeneca UK Ltd, AusDiagnostics, Basilea Pharmaceutica, Becton Dickinson Diagnostics, bioMérieux, Bio-Rad Laboratories, BSAC, Cepheid, Check-Points B.V., UK Department of Health, Enigma Diagnostics, Food Standards Agency, GlaxoSmithKline Services Ltd, Helperby Therapeutics, Henry Stewart Talks, IHMA Ltd, Innovate UK, Integra holdings, MSD, Meiji Seika Pharma Co. Ltd, Mobidiag, Momentum Biosciences Ltd, Neem Biotech, Nordic Pharma Ltd, Norgine Pharmaceuticals, Paratek Pharmaceuticals, Roche, Smith & Nephew UK Ltd, Shionogi & Co. Ltd, Trius Therapeutics, VenatoRx Pharmaceuticals and Wockhardt Ltd. C.H. was employed by Una Health Limited and QuantuMDx Group Limited during the period of data analysis and manuscript preparation. M.A. is a Trustee of the BSAC Council and is employed by MSD (UK) Limited, London, UK. M.A. has shareholdings in Merck & Co., Inc., Rahway, NJ, USA. C.L. is a Trustee of the BSAC Council and is employed by Shionogi B.V. B.J.P. is a consultancy: GenPax. D.M.L. is in Advisory Boards or *ad hoc* consultancy: AdjuTec, AstraZeneca, Centauri, GenPax, GSK, Lipovation, Meiji, Paion, Shionogi, Sumitovant, Thermo Fisher and Wockhardt; Paid lectures—bioMérieux, Pfizer, Shionogi and Zuellig Pharma. Relevant shareholdings or options—GenPax, GSK, Merck & Co Inc., and Revvity amounting to less than 10% of portfolio value. D.M.L. also has shareholdings in Arecor, Genincode, Probiotix, Rua Life Sciences, SkinBiotherapeutics and VericiDx (all with research/products pertinent to medicines or diagnostics) through Enterprise Investment Schemes, but has no authority to trade these holdings directly. All other authors have nothing to declare.

## Supplementary data

Supplementary Tables S1–S16, Figures S1–S4, and Appendix are available as Supplementary data at *JAC*  Online.

## References

[1] Anon . English Surveillance Programme for Antimicrobial Utilisation and Resistance (ESPAUR) report 2020 to 2021. 2020: 209. https://webarchive.nationalarchives.gov.uk/ukgwa/20221020175458/https://www.gov.uk/government/publications/english-surveillance-programme-antimicrobial-utilisation-and-resistance-espaur-report

[2] Wilson J, Elgohari S, Livermore DM et al Trends among pathogens reported as causing bacteraemia in England, 2004–2008. Clin Microbiol Infect 2011; 17: 451–8. 10.1111/j.1469-0691.2010.03262.x 20491834

[3] Bryan CS . Clinical implications of positive blood cultures. Clin Microbiol Rev 1989; 2: 329–53. 10.1128/CMR.2.4.329 2680055 PMC358128

[4] Johnson AP, Davies J, Guy R et al Mandatory surveillance of methicillin-resistant *Staphylococcus aureus* (MRSA) bacteraemia in England: the first 10 years. J Antimicrob Chemother 2012; 67: 802–9. 10.1093/jac/dkr561 22223229

[5] Pearson A, Chronias A, Murray M. Voluntary and mandatory surveillance for methicillin-resistant *Staphylococcus aureus* (MRSA) and methicillin-susceptible *S. aureus* (MSSA) bacteraemia in England. J Antimicrob Chemother 2009; 64: i11–7. 10.1093/jac/dkp260 19675013

[6] Izurieta P, Bahety P, Adegbola R et al Public health impact of pneumococcal conjugate vaccine infant immunization programs: assessment of invasive pneumococcal disease burden and serotype distribution. Expert Rev Vaccines 2018; 17: 479–93. 10.1080/14760584.2018.1413354 29241390

[7] Miller E, Andrews NJ, Waight PA et al Herd immunity and serotype replacement 4 years after seven-valent pneumococcal conjugate vaccination in England and Wales: an observational cohort study. Lancet Infect Dis 2011; 11: 760–8. 10.1016/S1473-3099(11)70090-1 21621466

[8] Horner C, Mushtaq S, Allen M et al Replacement of *Enterococcus faecalis* by *Enterococcus faecium* as the predominant enterococcus in UK bacteraemias. JAC-Antimicrob Resist 2021; 3: dlab185. 10.1093/jacamr/dlab185 34909690 PMC8664539

[9] Livermore DM . Fourteen years in resistance. Int J Antimicrob Agents 2012; 39: 283–94. 10.1016/j.ijantimicag.2011.12.012 22386741

[10] Tebano G, Zaghi I, Baldasso F et al Antibiotic resistance to molecules commonly prescribed for the treatment of antibiotic-resistant gram-positive pathogens: what is relevant for the clinician? Pathog Basel Switz 2024; 13: 88. 10.3390/pathogens13010088 PMC10819222 38276161

[11] Allen M, Reynolds R, Mushtaq S et al The British Society for Antimicrobial Chemotherapy Resistance Surveillance Project: methods and limitations. J Antimicrob Chemother 2025; 80 (Suppl 4): iv7–iv21. 10.1093/jac/dkn348 18819978

[12] Bischoff D. BLINDSCHEMES: Stata module to provide graph schemes sensitive to color vision deficiency. Statistical Software Components S458251, Boston College Department of Economics, revised 07 Aug 2020. https://ideas.repec.org/cgi-bin/refs.cgi.

[13] Paranthaman K, Wilson A, Verlander N et al Trends in coagulase-negative staphylococci (CoNS), England, 2010–2021. Access Microbiol 2023; 5: acmi000491.v3. 10.1099/acmi.0.000491.v3 PMC10323795 37424540

[14] Hawkey PM . Pre-clinical experience with daptomycin. J Antimicrob Chemother 2008; 62: iii7–14. 10.1093/jac/dkn367 18829726

[15] Farrell DJ, Castanheira M, Chopra I. Characterization of global patterns and the genetics of fusidic acid resistance. Clin Infect Dis 2011; 52: S487–92. 10.1093/cid/cir164 21546625

[16] Horner C, Mushtaq S, Livermore DM; BSAC Resistance Surveillance Standing Committee. Activity of ceftaroline versus ceftobiprole against staphylococci and pneumococci in the UK and Ireland: analysis of BSAC surveillance data. J Antimicrob Chemother 2020; 75: 3239–43. 10.1093/jac/dkaa306 32728710

[17] Jones RN . Microbiological features of vancomycin in the 21st century: minimum inhibitory concentration creep, bactericidal/static activity, and applied breakpoints to predict clinical outcomes or detect resistant strains. Clin Infect Dis 2006; 42: S13–24. 10.1086/491710 16323115

[18] Leclercq R, Dutka-Malen S, Duval J et al Vancomycin resistance gene *vanC* is specific to *Enterococcus gallinarum*. Antimicrob Agents Chemother 1992; 36: 2005–8. 10.1128/AAC.36.9.2005 1416893 PMC192426

[19] Anon . EUCAST: Clinical breakpoints and dosing of antibiotics. https://www.eucast.org/clinical_breakpoints/.

[20] Herrera-Hidalgo L, Fernández-Rubio B, Luque-Márquez R et al Treatment of *Enterococcus faecalis* infective endocarditis: a continuing challenge. Antibiot Basel Switz 2023; 12: 704. 10.3390/antibiotics12040704 PMC10135260 37107066

[21] Horner C, Reynolds R, Mushtaq S et al Trends of serotypes and resistance among *Streptococcus pneumoniae* in the UK and Ireland (1999–2019). J Antimicrob Chemother 2025; 80 (Suppl 4): iv72–iv86. 10.1093/jac/dkaf253 PMC12556646 41140271

[22] Doumith M, Mushtaq S, Martin V et al Genomic sequences of *Streptococcus agalactiae* with high-level gentamicin resistance, collected in the BSAC bacteraemia surveillance. J Antimicrob Chemother 2017; 72: 2704–7. 10.1093/jac/dkx207 29091185

[23] Liebowitz LD . MRSA burden and interventions. Int J Antimicrob Agents 2009; 34: S11–3. 10.1016/S0924-8579(09)70551-5 19596108

[24] Ellington MJ, Hope R, Livermore DM et al Decline of EMRSA-16 amongst methicillin-resistant *Staphylococcus aureus* causing bacteraemias in the UK between 2001 and 2007. J Antimicrob Chemother 2010; 65: 446–8. 10.1093/jac/dkp448 20035019

[25] Anon . European antimicrobial resistance surveillance network (EARS-net). Eur Cent Dis Prev Control. https://www.ecdc.europa.eu/en/about-us/partnerships-and-networks/disease-and-laboratory-networks/ears-net.

[26] Ladhani SN, Collins S, Djennad A et al Rapid increase in non-vaccine serotypes causing invasive pneumococcal disease in England and Wales, 2000–17: a prospective national observational cohort study. Lancet Infect Dis 2018; 18: 441–51. 10.1016/S1473-3099(18)30052-5 29395999

[27] Clarke SC, Scott KJ, McChlery SM. Erythromycin resistance in invasive serotype 14 pneumococci is highly related to clonal type. J Med Microbiol 2004; 53: 1101–3. 10.1099/jmm.0.45737-0 15496387

[28] Sheppard C, Fry NK, Mushtaq S et al Rise of multidrug-resistant non-vaccine serotype 15A *Streptococcus pneumoniae* in the United Kingdom, 2001 to 2014. Euro Surveill 2016; 21: 30423. 10.2807/1560-7917.ES.2016.21.50.30423 28006650 PMC5291132

[29] O’Neill AJ, Larsen AR, Skov R et al Characterization of the epidemic European fusidic acid-resistant impetigo clone of *Staphylococcus aureus*. J Clin Microbiol 2007; 45: 1505–10. 10.1128/JCM.01984-06 17344365 PMC1865894

[30] Hung W-C, Chen H-J, Lin Y-T et al Skin commensal staphylococci may act as reservoir for fusidic acid resistance genes. PLoS One 2015; 10: e0143106. 10.1371/journal.pone.0143106 26581090 PMC4651549

[31] Toleman MS, Reuter S, Jamrozy D et al Prospective genomic surveillance of methicillin-resistant *Staphylococcus aureus* (MRSA) associated with bloodstream infection, England, 1 October 2012 to 30 September 2013. Euro Surveill 2019; 24: 1800215. 10.2807/1560-7917.ES.2019.24.4.1800215 30696529 PMC6351993

[32] Lee JYH, Monk IR, Gonçalves da Silva A et al Global spread of three multidrug-resistant lineages of *Staphylococcus epidermidis*. Nat Microbiol 2018; 3: 1175–85. 10.1038/s41564-018-0230-7 30177740 PMC6660648

[33] Woodford N, Reynolds R, Turton J et al Two widely disseminated strains of *Enterococcus faecalis* highly resistant to gentamicin and ciprofloxacin from bacteraemias in the UK and Ireland. J Antimicrob Chemother 2003; 52: 711–4. 10.1093/jac/dkg408 12917231

[34] Raven KE, Reuter S, Gouliouris T et al Genome-based characterization of hospital-adapted *Enterococcus faecalis* lineages. Nat Microbiol 2016; 1: 15033. 10.1038/nmicrobiol.2015.33 27572164

[35] Barnett TC, Bowen AC, Carapetis JR. The fall and rise of group A *Streptococcus* diseases. Epidemiol Infect 2019; 147: e4. 10.1017/S0950268818002285 PMC6518539 30109840

[36] Gemmell CG . Glycopeptide resistance in *Staphylococcus aureus*: is it a real threat? J Infect Chemother Off J Jpn Soc Chemother 2004; 10: 69–75. 10.1007/s10156-004-0307-5 15160298

[37] Woodford N . Glycopeptide-resistant enterococci: a decade of experience. J Med Microbiol 1998; 47: 849–62. 10.1099/00222615-47-10-849 9788808

[38] van Hal SJ, Willems RJL, Gouliouris T et al The global dissemination of hospital clones of *Enterococcus faecium*. Genome Med 2021; 13: 52. 10.1186/s13073-021-00868-0 33785076 PMC8008517

