# Supplementary material for: Antimicrobial resistance among Gram-positive agents of bacteraemia in the UK and Ireland: trends from 2001 to 2019
Source: J Antimicrob Chemother. 2025 Oct 27;80(Suppl 4):iv22–35. doi: 10.1093/jac/dkaf249 (PMC12555346; doi:10.1093/jac/dkaf249)
Supplement: dkaf249_Supplementary_Data [file dkaf249_supplementary_data.pdf]

Antimicrobial resistance among Gram-positive agents of bacteraemia in the UK and Ireland: trends from 2001 to 2019

SUPPLEMENTARY INFORMATION

Methods for the BSAC Resistance Surveillance Project are described in detail in a companion paper.[1] This details also the UKHSA (UK Health Security Agency) bacteraemia surveillance, to which the BSAC data are cross-related.[2,3] Breakpoints and ECOFFs (Epidemiological Cut-offs) listed in Tables S4–S9 are from EUCAST tables of breakpoints (v12.0) and related table guidance at the time of analysis (https://www.eucast.org),[4] specifically:

European Committee on Antimicrobial Susceptibility Testing. **Breakpoint tables** for interpretation of MICs and zone diameters. Version 12.0, valid from 2022-01-01. https://www.eucast.org; https://www.eucast.org/fileadmin/src/media/PDFs/EUCAST_files/Breakpoint_tables/v_12.0_Breakpoint_Tables.pdf. Accessed 20 January 2025

European Committee on Antimicrobial Susceptibility Testing. Guidance document: **EUCAST breakpoints in brackets** 1 December, 2021. Available at: https://www.eucast.org/fileadmin/src/media/PDFs/EUCAST_files/Guidance_documents/Breakpoints_in_brackets.pdf /. Accessed 13 January 2025.

European Committee on Antimicrobial Susceptibility Testing. Data from the EUCAST MIC distribution website. https://www.eucast.org/**mic_and_zone_distributions_and_ecoffs**; https://mic.eucast.org/; https://mic.eucast.org/search/.

Contents

BSAC bacteraemia resistance surveillance – isolate collection and testing 3

Table S1. Isolate collection quotas and targets – BSAC Gram-positive bacteraemia surveillance 3

Table S2. Actual numbers of Gram-positive isolates tested, and centres contributing by year – BSAC bacteraemia surveillance 3

Data amendments and exclusions 4

Table S3. Data excluded after collection, based on evidence of flawed testing (BSAC surveillance)...... 4

Tables S4a & S4b. Numbers of Gram-positive isolates tested by organism subgroup and year (BSAC surveillance) 5

Table S4a Staphylococci: number of isolates tested by year (BSAC surveillance) 5

Table S4b. Enterococci and streptococci: number of isolates tested (BSAC surveillance) 6

Table S5. *S. aureus*: antibiotics analysed – years included, N of isolates, resistance breakpoints and mode MICs (BSAC surveillance).......... 7

Table S6a. Coagulase-negative staphylococci: antibiotics analysed – years included, N of isolates, resistance breakpoints and mode MICs (BSAC data) 8

Table S6b. Coagulase-negative staphylococci by species: antibiotics analysed – years included, N of isolates, resistance breakpoints and mode MICs (BSAC data).......... 9

Table S7a. Enterococci: antibiotics analysed – years included, N of isolates, resistance breakpoints and mode MICs (BSAC data) 10

Table S7b. Enterococci other than *E. faecalis*/*E. faecium* by species: antibiotics analysed – N of isolates, resistance breakpoints and mode MICs (BSAC data) 10

Table S8. *S. pneumoniae*: antibiotics analysed – years included, N of isolates, resistance breakpoints and mode MICs (BSAC data) 11

Table S9a. α-Haemolytic streptococci: antibiotics analysed – years included, N of isolates, resistance breakpoints and mode MIC (BSAC data) 11

Table S9b. α-Haemolytic streptococci by species group: antibiotics analysed – N of isolates, resistance breakpoints and mode MICs (BSAC data) 12

Table S10. β-Haemolytic streptococci: antibiotics analysed – years included, N of isolates, resistance breakpoints and mode MICs (BSAC data) 13

BSAC bacteraemia resistance surveillance – patient characteristics 14

Table S11. Proportion of male patients by organism sub-group 14

Table S12. Patient age: summary measures by organism group 15

Figure S1. Patient age: histograms and trends by organism group 15

Figure S2. Trends in care setting by organism group 17

Table S13. Proportion of isolates from ICU patients, by organism group 19

Figure S3. Trends in the proportion of isolates from ICU patients by organism group 20

Tables S14. Source of infection: top three or all ≥10% of isolates, by organism 21

UKHSA bacteraemia surveillance – routine data, England only 22

Table S15. Number of reported bacteraemias due to Gram-positive bacteria (UKHSA surveillance, routine data) 22

Table S16. Staphylococcal bacteraemias, by methicillin status (UKHSA surveillance, routine data) 23

Figure S4. Numbers of reported Gram-positive bacteraemias with organisms included in BSAC surveillance (UKHSA surveillance, routine data). 23

Figure S5 Trends in percentage of bacteraemias reported to UKHSA with antimicrobial susceptibility test results, by organism group (UKHSA surveillance, routine data) 24

References 26

APPENDIX – MIC distributions 26

Staphylococci 27

Enterococci 32

S. pneumoniae 34

α- and non-Haemolytic streptococci 35

β-Haemolytic streptococci 36

β-Haemolytic streptococci 36

BSAC bacteraemia resistance surveillance – isolate collection and testing

Table S1. Isolate collection quotas and targets – BSAC Gram-positive bacteraemia surveillance

Annual collection periods[1]	Target N of centres	*S. aureus* Quota/lab	*S. aureus* Target	All other collection groups[2] Quota/lab	All other collection groups[2] Target
2001–2007	25	10	250	10	250
2008–2009	25	20	500	10	250
2010–2015	40	14	560	7	280
2016–2019	25	20	500	10	250

[1] January–December

[2] Coagulase-negative staphylococci (CoNS), enterococci, *S. pneumoniae*, other α-haemolytic streptococci, β-haemolytic streptococci.

Collections per participating centre per year averaged >90% of the requested quota of Gram-positive isolates for *S. aureus* (95%) and enterococci (92%), 88–90% for *S. pneumoniae* and β-haemolytic streptococci, 81% for coagulase-negative streptococci (CoNS) and 72% for α-haemolytic streptococci.

Table S2. Actual numbers of Gram-positive isolates tested, and centres contributing by year – BSAC bacteraemia surveillance

Year	N of Centres[1]	N of isolates					
		Staphylococci		Enterococci	Streptococci		
		S. aureus	coagulase-negative		*S. pneumoniae*	α-haemolytic	β-haemolytic
2001	25	238	209	222	227	160	215
2002	25	245	200	224	220	173	205
2003	25	235	212	245	239	169	232
2004	25	244	187	235	241	164	225
2005	25	244	203	243	230	180	227
2006	25	242	203	238	231	166	228
2007	25	245	181	224	216	169	223
2008	24	453	179	206	201	144	201
2009	25	470	205	215	211	156	225
2010	39	505	232	245	249	210	244
2011	38	516	223	235	230	191	237
2012	39	515	203	255	229	190	244
2013	39	522	214	255	235	207	258
2014	40	504	223	250	247	219	254
2015	40	514	213	251	244	215	248
2016	25	478	225	247	220	185	240
2017	25	453	219	225	208	198	220
2018	24	456	201	235	208	178	212
2019	24	475	222	236	215	220	234
Total	(81)	7554	3954	4486	4301	3494	4372

See Tables S4A and S4B for smaller groups and species within these collection groups e.g. *E. faecalis*, *S. mitis* group.

[1] Number of sites that actually contributed any Gram-positive isolates from bacteraemia in that year; not all necessarily contributed isolates of all organism groups.

Data amendments and exclusions

Five isolates of *S. aureus* recorded as *mecA*-negative but with discrepantly high MICs for oxacillin (≥8 mg/L) were noted during data review for this paper and were retested in 2024. Three (with MICs of 8 mg/L) were confirmed *mecA*-negative and are reported without amendment. One (MIC 128 mg/L) was confirmed *mecA*-negative; after further testing, its oxacillin MIC was revised to 1 mg/L. The fifth (MIC 128 mg/L) was found *mecA*-positive on retest and, accordingly, was reclassified as MRSA.

Table S3. Data excluded after collection, based on evidence of flawed testing (BSAC surveillance)

Antimicrobial	Year(s)	Organism(s)	Reason
Daptomycin	2019	Staphylococci<br>Enterococci<br>Streptococci	The MIC distribution was shifted upwards by one doubling dilution compared with other years (including for control strains of staphylococci), resulting in misleading overestimates of resistance.
Erythromycin	2003<br>2004	α-Haemolytic streptococci	In each of these years, around half the MICs across the whole range were shifted up by 2–3 doubling dilutions.
Tigecycline	2002<br>2003	All Gram-positive organisms	Optimum testing conditions were unclear in the earliest years, and MIC distributions for several organism groups were distorted compared with those of later years.

Tables S4a & S4b. Numbers of Gram-positive isolates tested by organism subgroup and year (BSAC surveillance)

All isolates were re-identified and tested at the Antimicrobial Resistance and Healthcare-Associated Infections Reference Unit (or its predecessor laboratory) at the UK Health Security Agency (UKHSA) in Colindale, London (previously Public Health Laboratory Service, Health Protection Agency, then Public Health England).

Table S4a Staphylococci: number of isolates tested by year (BSAC surveillance)

Year	*S. aureus*		CoNS		CoNS species					
	MSSA	MRSA	MSCoNS	MRCoNS	*S. epidermidis*	*S. hominis*	*S. haemolyticus*	*S. capitis*	Other named	No species ID
2001	135	103	22	187	137	17	23	8	6	18
2002	145	100	43	157	102	23	34	15	5	21
2003	140	95	38	174	126	21	27	30	2	6
2004	126	118	36	151	121	23	18	12	4	9
2005	156	88	51	152	129	23	21	7	4	19
2006	137	105	45	158	N/A	N/A	N/A	N/A	N/A	203
2007	159	86	40	141	N/A	N/A	N/A	N/A	N/A	181
2008	338	115	49	130	N/A	N/A	N/A	N/A	N/A	179
2009	366	104	49	156	N/A	N/A	N/A	N/A	N/A	205
2010	407	98	61	171	N/A	N/A	N/A	N/A	N/A	232
2011	446	70	71	152	N/A	N/A	N/A	N/A	N/A	223
2012	454	61	47	156	N/A	N/A	N/A	N/A	N/A	203
2013	460	62	63	151	144	23	18	13	16	0
2014	453	51	65	158	130	33	22	21	16	1
2015	473	41	62	151	137	26	24	13	13	0
2016	437	41	59	166	148	25	24	14	14	0
2017	424	29	69	150	147	33	18	15	5	1
2018	428	28	58	143	133	28	19	18	3	0
2019	449	26	65	157	142	37	24	12	7	0

N/A: not applicable (2006–12) because CoNS species were identified only in 2001–05 (by PCR of DNA encoding 16S ribosomal RNA) and 2013–19 (by MALDI-TOF).

Table S4b. Enterococci and streptococci: number of isolates tested (BSAC surveillance)

Year	Enterococci: species			α/non-Haemolytic streptococci: species groups					β-Haemolytic streptococci: groups		
	E. faecalis	*E. faecium*	other / unnamed	*S. mitis*	*S. anginosus*	*S. bovis*	*S. salivarius*	*S. mutans*	A	B	C/G
2001	151	68	3	97	36	18	8	1	91	67	57
2002	149	70	5	89	44	22	13	5	76	70	59
2003	156	74	15	103	30	17	17	2	110	68	54
2004	139	80	16	102	24	22	14	2	103	62	60
2005	138	90	15	107	31	21	18	3	88	65	74
2006	142	81	15	102	30	21	12	1	85	82	61
2007	118	90	16	91	38	24	12	4	72	94	57
2008	117	73	16	81	31	14	15	3	67	78	56
2009	130	75	10	83	31	24	15	3	71	89	65
2010	125	108	12	127	35	24	18	6	78	105	61
2011	126	88	21	120	29	22	18	2	87	91	59
2012	128	115	12	105	41	17	22	5	69	85	90
2013	136	112	7	129	28	23	20	7	108	84	66
2014	122	115	13	131	41	24	18	3	93	85	76
2015	126	104	21	121	31	33	23	7	99	71	78
2016	105	127	15	113	37	18	14	3	69	91	80
2017	101	120	4	113	36	22	24	3	64	77	79
2018	111	115	9	103	37	17	19	2	69	68	75
2019	108	119	9	115	54	27	18	6	61	68	105

See Table S2 for *S. pneumoniae*.
Not shown: 2 isolates of α/non-haemolytic streptococci not identified to species-group level.

Table S5. *S. aureus*: antibiotics analysed – years included, N of isolates, resistance breakpoints and mode MICs (BSAC surveillance)

Antimicrobial	Years included	N of years	Break-point R > mg/L	MSSA		MRSA	
				N of isolates	Mode MIC mg/L	N of isolates	Mode MIC mg/L
Ceftaroline	2008, 2013, 2017–19	5	2	2099	0.25	260	1
Ceftobiprole	2003–10, 2012–19	16	2	5407	0.5	1148	2
Ciprofloxacin	2001–19	19	1	6133	0.5‡	1421	128‡
Clindamycin[1]	2001–19	19	0.25	6133	0.12‡	1421	0.12‡
Daptomycin[2]	2003, 2005–07, 2009–10	6	1	1365	0.5	576	0.5
Erythromycin	2001–19	19	2	6133	0.5‡	1421	≥256‡
Fusidic acid	2003–19	17	1	5853	0.12‡	1218	0.12‡
Gentamicin[3]	2001–19	19	2	6133	0.25	1421	0.25‡
Linezolid	2001–13, 2019	14	4	3918	2	1231	2
Minocycline	2002–15	14	0.5	4260	0.12‡	1194	0.12‡
Mupirocin[4]	2007–19	13	1	5294	0.25‡	812	0.25‡
Oxacillin[5]	2001–19	19	2	6133	0.25	1421	≥256
Penicillin	2001–15	15	0.12	4395	≥128‡	1297	≥128
Rifampicin	2003–19	17	0.06	5853	0.008‡	1218	0.008‡
Tedizolid	2015–19	5	0.5	2211	0.25	165	0.25
Teicoplanin	2001–19	19	2	6133	1	1421	1
Tetracycline	2001–19	19	2	6133	0.5‡	1421	0.5‡
Tigecycline	2004–13	10	0.5	3049	0.25	907	0.25
Trimethoprim[6]	2001–19	19	4	6133	0.5‡	1421	0.5‡
Vancomycin	2001–19	19	2	6133	1	1421	1

‡ Most frequent MIC, but distribution was clearly bi- or multi-modal: refer to plot in Appendix.

[1] Clindamycin resistance is reported at face value using this 2022 EUCAST breakpoint (MIC >0.25 mg/L). Inducible resistance was tested (2012–2019) with 4 mg/L erythromycin plus 0.5 mg/L clindamycin, the then breakpoint, so may be slightly underestimated relative to the lower 2022 breakpoint.

[2] Daptomycin breakpoint not validated for testing by agar dilution.

[3] Epidemiological cut-off (ECOFF) 'breakpoint-in-brackets', intended to distinguish isolates with acquired resistance mechanisms likely to undermine effectiveness when used as a synergist in combination therapy.

[4] ECOFF, for descriptive purposes. Mupirocin resistance was further assessed by PCR for *mupA*.

[5] Oxacillin was tested in all years, but MRSA was defined by its phenotypic resistance (MIC >2 mg/L) only in 2001–04. From 2005 onwards, MRSA was defined by PCR for *mecA*.

[6] Clinical breakpoint for uncomplicated UTI only. Trimethoprim is of limited relevance outside UTI, but co-trimoxazole is occasionally advocated for wider use in the NHS.(5)

Table S6a. Coagulase-negative staphylococci: antibiotics analysed – years included, N of isolates, resistance breakpoints and mode MICs (BSAC data)

Antimicrobial	Years included	N of years	Break-point R> mg/L	MSCoNS		MRCoNS	
				N of isolates	Mode MIC mg/L	N of isolates	Mode MIC mg/L
Ceftaroline	2008, 2013, 2017–19	5	x	304	0.06	731	0.25
Ceftobiprole[1]	2003–10, 2012–19	16	2	857	0.25	2465	1
Ciprofloxacin	2001–19	19	1	993	0.25[‡]	2961	0.25[‡]
Clindamycin[2]	2001–19	19	0.25	993	0.12[‡]	2961	0.12[‡]
Daptomycin[3]	2003, 2005–07, 2009–10	6	1	284	0.5	952	0.5
Erythromycin	2001–19	19	2	993	0.25[‡]	2961	≥256[‡]
Fusidic acid	2003–19	17	1	928	0.12[‡]	2617	0.12[‡]
Gentamicin[4]	2001–19	19	2	993	0.06[‡]	2961	≤0.25[‡]
Linezolid	2001–13, 2019	14	4	680	1	2193	1
Minocycline	2002–15	14	0.5	720	0.12	2158	0.5
Mupirocin[5]	2007–19	13	x	758	0.25[‡]	1982	0.25[‡]
Oxacillin[6]	2001–19	19	0.25	993	0.12	2961	≥256[‡]
Penicillin	2001–15	15	x	742	0.03[‡]	2345	≥128[‡]
Rifampicin	2003–19	17	0.06	928	0.015[‡]	2617	0.008[‡]
Tedizolid	2015–19	5	0.5	313	0.25	767	0.25
Teicoplanin	2001–19	19	4	993	4	2961	4
Tetracycline	2001–19	19	2	993	0.5	2961[‡]	2[‡]
Tigecycline	2004–13	10	0.5	512	0.25	1518	0.5
Trimethoprim[7]	2001–19	19	4	993	0.5	2961[‡]	≥256[‡]
Vancomycin	2001–19	19	4	993	2	2961	2

x No breakpoint or epidemiological cut-off (ECOFF) available.

‡ Most frequent MIC, but distribution was clearly bi- or multi-modal: refer to plot in Appendix and note species differences (Table S6b).

[1] *S. aureus* breakpoint applied unofficially for descriptive purposes.

[2] Clindamycin resistance is reported at face value using this 2022 EUCAST breakpoint (MIC >0.25 mg/L). Inducible resistance was tested (2012–2019) with 4 mg/L erythromycin plus 0.5 mg/L clindamycin, the then breakpoint, so may be slightly underestimated relative to the lower 2022 breakpoint.

[3] Daptomycin breakpoint not validated for testing by agar dilution.

[4] Gentamicin MIC >2 mg/L is the EUCAST 'breakpoint in brackets' for all staphylococci. It is intended to distinguish isolates with acquired resistance mechanisms likely to undermine synergy with active partner agents, but it does not match the epidemiological cut-off (ECOFF) for *S. epidermidis* (R>0.25); there are no designated ECOFFs for other *Staphylococcus* spp.

[5] ECOFF, for descriptive purposes. Mupirocin resistance was further assessed by PCR for *mupA*.

[6] Oxacillin was tested every year but MRCoNS was defined by its phenotypic resistance (MIC >2 mg/L) only in 2001–04. From 2005 onwards, MRCoNS was defined by PCR for *mecA*.

[7] Clinical breakpoint for uncomplicated UTI only. Trimethoprim is of limited relevance outside UTI, but co-trimoxazole is occasionally advocated for wider use in the NHS.[5]

Table S6b. Coagulase-negative staphylococci by species: antibiotics analysed – years included, N of isolates, resistance breakpoints and mode MICs (BSAC data)

Antimicrobial	Years included	N of years	Break-point R> mg/L	*S. epidermidis*		*S. hominis*		*S. haemolyticus*		*S. capitis*	
				N of isolates	Mode MIC mg/L	N of isolates	Mode MIC mg/L	N of isolates	Mode MIC mg/L	N of isolates	Mode MIC mg/L
Ceftaroline	2013; 2017–2019	4	x	566	0.25‡	121	0.12‡	79	2	58	0.06‡
Ceftobiprole[1]	2003–2005; 2013–2019	10	2	1,357	1	272	2‡	215	4	155	1‡
Ciprofloxacin	2001–2005; 2013–2019	12	1	1,596	0.25‡	312	0.12‡	272	128‡	178	0.25‡
Clindamycin[2]	2001–2005; 2013–2019	12	0.25	1,596	0.12‡	312	0.12‡	272	0.12‡	178	0.12‡
Daptomycin[3]	2003; 2005	2	1	255	0.5	44	0.25†	48	0.5†	37	0.5†
Erythromycin	2001–2005; 2013–2019	12	2	1,596	≥256‡	312	≥256‡	272	≥256‡	178	0.25‡
Fusidic acid	2003–2005; 2013–2019	10	1	1,357	0.12‡	272	16‡	215	0.12‡	155	0.12‡
Gentamicin[4]	2001–2005; 2013–2019	12	2	1,596	0.06‡	312	0.03‡	272	32‡	178	0.03‡
Linezolid	2001–2005; 2013; 2019	7	4	901	1	167	1	165	1	97	1
Minocycline	2002–2005; 2013–2015	7	0.5	889	0.5‡	172	0.12	164	0.5	111	0.12
Mupirocin[5]	2013–2019	7	x	981	0.25‡	205	0.25‡	149	0.25‡	106	0.25‡
Oxacillin[6]	2001–2005; 2013–2019	12	0.25	1,596	0.12‡	312	0.12‡	272	≥256‡	178	≥256‡
Penicillin	2001–2005; 2013–15	8	x	1,026	≥128‡	189	≥128‡	187	≥128‡	119	≥128‡
Rifampicin	2003–2005; 2013–2019	10	0.06	1,357	0.008‡	272	0.008‡	215	0.015‡	155	0.03‡
Tedizolid	2015–2019	5	0.5	707	0.25	149	0.25	109	0.25	72	0.25
Teicoplanin	2001–2005; 2013–2019	12	4	1,596	4	312	0.5	272	4	178	0.5
Tetracycline	2001–2005; 2013–2019	12	2	1,596	2‡	312	0.5‡	272	2‡	178	0.5‡
Tigecycline	2004–2005	2	0.5	394	0.5	69	0.12†	57	0.5†	32	0.25†
Trimethoprim[7]	2001–2005; 2013–2019	12	4	1,596	≥256	312	64	272	≥256	178	1
Vancomycin	2001–2005; 2013–2019	12	4	1,596	2	312	1	272	2	178	2

‡ Most frequent MIC, but distribution was clearly bi- or multi-modal.

[1–7] See Table S6a for antimicrobial notes.

N.B. CoNS were not identified to species level from 2006 to 2012.

Omitted: 79 isolates of other named species and 75 identified only to genus level in the periods when CoNS were tested for species identification.

x No breakpoint or epidemiological cut-off (ECOFF) available.

Caution (MIC modes): † result based on few (20–49); isolates **xx** result based on <20 isolates not shown.

Table S7a. Enterococci: antibiotics analysed – years included, N of isolates, resistance breakpoints and mode MICs (BSAC data)

Antimicrobial	Years included	N of years	Break-point R> mg/L	*E. faecalis*		*E. faecium*		Other/unnamed	
				N of isolates	Mode MIC mg/L	N of isolates	Mode MIC mg/L	N of isolates	Mode MIC mg/L
Ampicillin	2001–19	19	8	2428	1‡	1824	≥256‡	234	0.5‡
Ciprofloxacin	2001–15	15	x	2003	2‡	1343	≥256‡	197	1‡
Daptomycin	2003; 2005–07; 2009–10	6	x	809	0.5	518	1	83	0.5‡
Gentamicin[1]	2001–19	19	128	2428	≥4096‡	1824	≥4096‡	234	4‡
Linezolid	2001–13; 2019	14	4	1863	2	1243	2	172	2‡
Tedizolid	2015–19	5	x	551	0.5	585	0.5	58	0.5
Teicoplanin	2001–19	19	2	2428	0.25‡	1824	1‡	234	0.5‡
Tigecycline	2004–13	10	0.25	1299	0.12	912	0.06	140	0.06‡
Vancomycin	2001–19	19	4	2428	2‡	1824	1‡	234	1‡

‡ Most frequent MIC, but distribution was clearly bi- or multi-modal: refer to plots in Appendix for *E. faecalis* and *E. faecium* and note species differences (Table S7b) for others.
[1] Gentamicin screening breakpoint for high-level acquired aminoglycoside resistance (HLAR).
See Table 7b for breakdown by five most numerous other named species of enterococci.

Table S7b. Enterococci other than *E. faecalis/E. faecium* by species: antibiotics analysed – N of isolates, resistance breakpoints and mode MICs (BSAC data)

Antimicrobial	N of years	Break-point R> mg/L	*E. durans*		*E. gallinarum*		*E. avium*		*E. casseliflavus*		*E. raffinosus*	
			N of isolates	Mode‡ MIC mg/L	N of isolates	Mode‡ MIC mg/L	N of isolates	Mode‡ MIC mg/L	N of isolates	Mode‡ MIC mg/L	N of isolates	Mode‡ MIC mg/L
Ampicillin	19	8	75	1	33	1	30	0.5	24	0.5	18	xx
Ciprofloxacin	15	x	67	1‡	28	2‡	21	2‡	21	2	6	xx
Daptomycin	6	x	23	2	8	xx	5	xx	10	xx	0	xx
Gentamicin[1]	19	128	75	4	33	4	30	1	24	4	18	xx
Linezolid	14	4	43	2	27	2	22	2	21	2	5	xx
Tedizolid	5	x	23	0.5	6	xx	13	xx	3	xx	13	xx
Teicoplanin	19	2	75	0.5	33	0.5	30	0.5	24	1	18	xx
Tigecycline	10	0.25	39	0.06	21	0.12	12	xx	16	xx	4	xx
Vancomycin	19	4	75	1	33	8	30	1	24	4	18	xx

See Table 7A for *E. faecalis*, *E. faecium* and total of other/unnamed species, and for years included.
[1] Gentamicin screening breakpoint for high-level acquired aminoglycoside resistance (HLAR).
Omitted: 52 isolates not identified to species level and 2 *E. hirae*
Caution (MIC modes): all estimates are based on small numbers of isolates (maximum 75); **xx** result based on <20 isolates not shown.
[‡ Markers for bi- or multi-modality omitted: small numbers make detection of sub-populations unreliable.]

Table S8. *S. pneumoniae*: antibiotics analysed – years included, N of isolates, resistance breakpoints and mode MICs (BSAC data)

Antimicrobial	Years included	N of years	Break-point R> mg/L	*S. pneumoniae* N of isolates	*S. pneumoniae* Mode MIC mg/L
Amoxicillin[1]	2001–19	19	1	4301	0.015‡
Cefotaxime	2001–19	19	2	4301	0.015‡
Ceftaroline	2008; 2013; 2017–19	5	0.25	1067	0.008
Ceftobiprole	2004–10; 2012–19	15	0.5	3385	0.015‡
Clindamycin[2]	2001–19	19	0.5	4301	0.12‡
Erythromycin	2001–19	19	0.5	4301	0.12‡
Imipenem	2001–13; 2015–19	18	2	4054	0.004‡
Linezolid	2001–13; 2019	14	2	3174	1
Meropenem	2006–08; 2014–19	9	2	1990	0.008‡
Penicillin[3]	2001–19	19	2	4301	0.015‡
Teicoplanin	2001–19	19	2	4301	0.06
Tetracycline	2001–19	19	2	4301	0.25‡
Vancomycin	2001–19	19	2	4301	0.5

‡ Most frequent MIC, but distribution was clearly bi- or multi-modal: refer to plot in Appendix.
[1] Amoxicillin has no i.v. breakpoint, but this oral breakpoint matches the i.v. breakpoint for ampicillin.
[2] Clindamycin resistance is reported at face value using this 2022 EUCAST breakpoint (MIC >0.5 mg/L). Inducible resistance was not tested for *S. pneumoniae.*
[3] Isolates with penicillin MIC >0.06 mg/L but ≤2 mg/L are classified I ("susceptible, increased exposure").

Table S9a. α-Haemolytic streptococci: antibiotics analysed – years included, N of isolates, resistance breakpoints and mode MIC (BSAC data)

Antimicrobial	Years included	N of years	Break-point R> mg/L	All α-haemolytic streptococci N of isolates	All α-haemolytic streptococci Mode MIC mg/L
Cefotaxime	2001–19	19	0.5	3494	0.12
Ceftobiprole	2004–10; 2012–19	15	x	2801	0.03‡
Clindamycin[1]	2001–19	19	0.5	3494	0.06‡
Daptomycin	2003; 2005–07; 2009–10	6	x	1050	0.5
Erythromycin[2,3]	2001–02; 2005–19	17	0.25	3161	0.12‡
Gentamicin[4]	2001–19	19	128	3494	8
Linezolid	2001–13; 2019	14	x	2499	1
Penicillin	2001–19	19	2	3494	0.06
Tedizolid[5]	2015–19	5	0.5	996	0.25
Teicoplanin	2001–19	19	2	3494	0.12
Tetracycline[2]	2001–19	19	2	3494	0.5‡
Tigecycline	2004–13	10	x	1777	0.06
Vancomycin	2001–19	19	2	3494	0.5

‡ Most frequent MIC, but distribution was clearly bi- or multi-modal: refer to plot in Appendix.
See Table 9b for a breakdown by species groups.
[1] Clindamycin resistance is reported at face value using this 2022 EUCAST breakpoint (MIC >0.5 mg/L). Inducible resistance was tested (2012–19) with 4 mg/L erythromycin plus 0.5 mg/L clindamycin.
[2] Tentative epidemiological cut-offs (ECOFFs); higher values suggest acquired resistance mechanisms.
[3] Erythromycin ECOFF published for *S. anginosus* and S. *oralis*,(4) applied here for all groups.
[4] Gentamicin screening breakpoint for high-level acquired aminoglycoside resistance (HLAR).
[5] Tedizolid breakpoint applies to *S. anginosus* group only.

Table S9b. α-Haemolytic streptococci by species group: antibiotics analysed – N of isolates, resistance breakpoints and mode MICs (BSAC data)

Antimicrobial	N of years	Break-point R> mg/L	*S. mitis* group		*S. anginosus* group		*S. bovis* group		*S. salivarius* group		*S. mutans* group	
			N of isolates	Mode MIC mg/L	N of isolates	Mode MIC mg/L	N of isolates	Mode MIC mg/L	N of isolates	Mode MIC mg/L	N of isolates	Mode MIC mg/L
Cefotaxime	19	0.5	2032	0.06	664	0.12	410	0.12‡	318	0.03‡	68	0.03
Ceftobiprole	15	x	1623	0.03	525	0.12	331	0.015	262	0.015	58	0.015
Clindamycin[1]	19	0.5	2032	0.06‡	664	0.06‡	410	0.12‡	318	0.06‡	68	0.06
Daptomycin	6	x	613	0.5	195	0.5	131	0.12	92	0.25	19	xx
Erythromycin[2,3]	17	0.25	1827	0.12‡	610	0.12‡	371	0.12‡	287	0.12‡	64	0.12‡
Gentamicin[4]	19	128	2032	8‡	664	16	410	8	318	16	68	4
Linezolid	14	x	1451	1	482	1	296	2	220	1	50	1
Penicillin	19	2	2032	0.06	664	0.03	410	0.06	318	0.06‡	68	0.03
Tedizolid[5]	5	0.5	565	0.25	195	0.25	117	0.25	98	0.25	21	0.25†
Teicoplanin	19	2	2032	0.12	664	0.12	410	0.25	318	0.25	68	0.5
Tetracycline[2]	19	2	2032	0.5‡	664	0.5‡	410	128‡	318	0.5‡	68	0.5‡
Tigecycline	10	x	1047	0.06	318	0.06	212	0.12	164	0.06	36	0.12†
Vancomycin	19	2	2032	0.5‡	664	1‡	410	0.5	318	0.5	68	1

‡ Most frequent MIC, but distribution was clearly bi- or multi-modal.

[1–5] See Table S9a for antimicrobial notes, and for years included.

Omitted: two isolates of unidentified species group.

Caution (MIC modes, *S. mutans* group): **†** result based on small number of isolates (20–49); **xx** result based on <20 isolates not shown.

MIC distributions are commonly bi- or multi-modal, reflecting subpopulations with different levels of resistance/susceptibility. The modes shown typically correspond to the (usually more numerous) susceptible subpopulation, but will relate to the resistant subpopulation if it is sufficiently dominant and concentrated (e.g. *S. bovis*/tetracycline, 70% R).

Table S10. β-Haemolytic streptococci: antibiotics analysed – years included, N of isolates, resistance breakpoints and mode MICs (BSAC data)

Antimicrobial	Years included	N of years	Break-point R> mg/L	All β-haemolytic		Group A		Group B		Group C/G	
				N of isolates	Mode MIC mg/L	N of isolates	Mode MIC mg/L	N of isolates	Mode MIC mg/L	N of isolates	Mode MIC mg/L
Cefotaxime	2001–15	15	x	3466	0.015	1297	≤0.008	1196	0.06	973	0.015
Ceftobiprole	2004–10; 2012–19	15	x	3483	0.008	1196	0.008	1204	0.03	1083	0.008
Clindamycin[1]	2001–19	19	0.5	4372	0.06‡	1560	0.06‡	1500	0.12‡	1312	0.06‡
Daptomycin	2003; 2005–07; 2009–10	6	1	1379	0.06‡	504	0.06	503	0.25	372	0.06
Erythromycin	2001–19	19	0.5	4372	0.06‡	1560	0.06‡	1500	0.06‡	1312	0.06‡
Gentamicin	2001–15	15	x	3466	4	1297	4	1196	8	973	4
Linezolid	2001–13; 2019	14	2	3198	2	1166	1	1108	2	924	2
Penicillin	2001–19	19	0.25	4372	0.008‡	1560	0.008	1500	0.03	1312	0.008
Tedizolid	2015–19	5	0.5	1154	0.25	362	0.25	375	0.25	417	0.25
Teicoplanin	2001–19	19	2	4372	0.12	1560	0.12	1500	0.25	1312	0.12
Tetracycline	2001–19	19	2	4372	0.5‡	1560	0.5‡	1500	64‡	1312	0.5‡
Tigecycline	2004–13	10	0.12	2312	0.12	828	0.06	835	0.12	649	0.12
Vancomycin	2001–19	19	2	4372	0.5	1560	0.5	1500	0.5	1312	0.5

‡ Most frequent MIC, but distribution was clearly bi- or multi-modal: refer to plots in Appendix.

[1] Clindamycin resistance is reported at face value using this 2022 EUCAST breakpoint (MIC >0.5 mg/L). Inducible resistance was tested (2012–2019) with 4 mg/L erythromycin plus 0.5 mg/L clindamycin.

BSAC bacteraemia resistance surveillance – patient characteristics

Sex

In general, and as noted previously for UKHSA data, there was a preponderance of male patients ranging from 52% (β-haemolytic streptococci) to 63% (*S. aureus*). There were also clear differences between organism subgroups.

Table S11. Proportion of male patients by organism sub-group

Gram-positive		
Organism group	N[1]	Male, %
MSSA	6120	62.7
MRSA	1417	65.3
S. aureus, all	7537	63.2
MSCoNS	991	56.2
MRCoNS	2942	55.6
CoNS, all	3933	55.8
*S. epidermidis**	1589	55.2
*S. hominis**	311	46.3
*S. haemolyticus**	270	66.7
*S. capitis**	176	60.2
other named CoNS*†	94	56
unspecified CoNS*†	71	48
CoNS, all	3933	55.8
E. faecalis	2423	63.8
E. faecium	1824	56.4
Enterococcus: other/spp.	234	53.4
Enterococcus, all	4481	60.2
S. pneumoniae	4294	53.2
S. mitis group	2029	57.3
S. anginosus group	664	65.1
S. bovis group	409	56.7
S. salivarius group	316	56.3
S. mutans group†	68	71
α-Haemolytic strep, all	3488	58.9
β-Haemolytic strep: A	1559	55.3
β-Haemolytic strep: B	1492	47.1
β-Haemolytic strep: C/G	1310	52.1
β-Haemolytic strep, all	4361	51.5

Not shown: two unidentified α-haemolytic streptococci

[1] Number of isolates with sex data for the source patient (Missing: 67/28,161 = 0.2%)

* CoNS species were determined from 2001–05 and 2013–19 only; they were not identified from 2006–12.

† Caution: 51–100 isolates only; imprecise estimates.

Age

All organism groups had infants under one year old as a distinct subgroup of source patients, largest for CoNS and β-haemolytic streptococci (both over 10% overall). Considering species and subgroups of CoNS and streptococci, the proportions of infants were particularly high among patients with bacteraemias caused by *S. capitis* (31%), *S. epidermidis* (10%), group B streptococci (29%) and *S. salivarius* (13%).

Table S12. Patient age: summary measures by organism group

Organism group	N[1]	Quartiles, years			Isolates in age group shown, %		
		Q1	Q2	Q3	<1 year	≥65 years	≥80 years
S. aureus	7515	46	65	78	3.5	51.2	21.8
CoNS	3936	30	55	68	10.9	31.3	7.4
Enterococci	4464	52	68	78	4.0	56.9	22.1
S. pneumoniae	4280	44	65	79	3.1	50.1	23.2
α-Haemolytic streptococci	3477	38	61	76	4.6	45.1	17.2
β-Haemolytic streptococci	4351	33	62	78	10.8	46.9	21.9

[1] Number of isolates with age data. (Missing: 67/28,161 = 0.2%)

Patients aged ≥80 years are an older subset of those who are ≥65 years old.

Figure S1. Patient age: histograms and trends by organism group

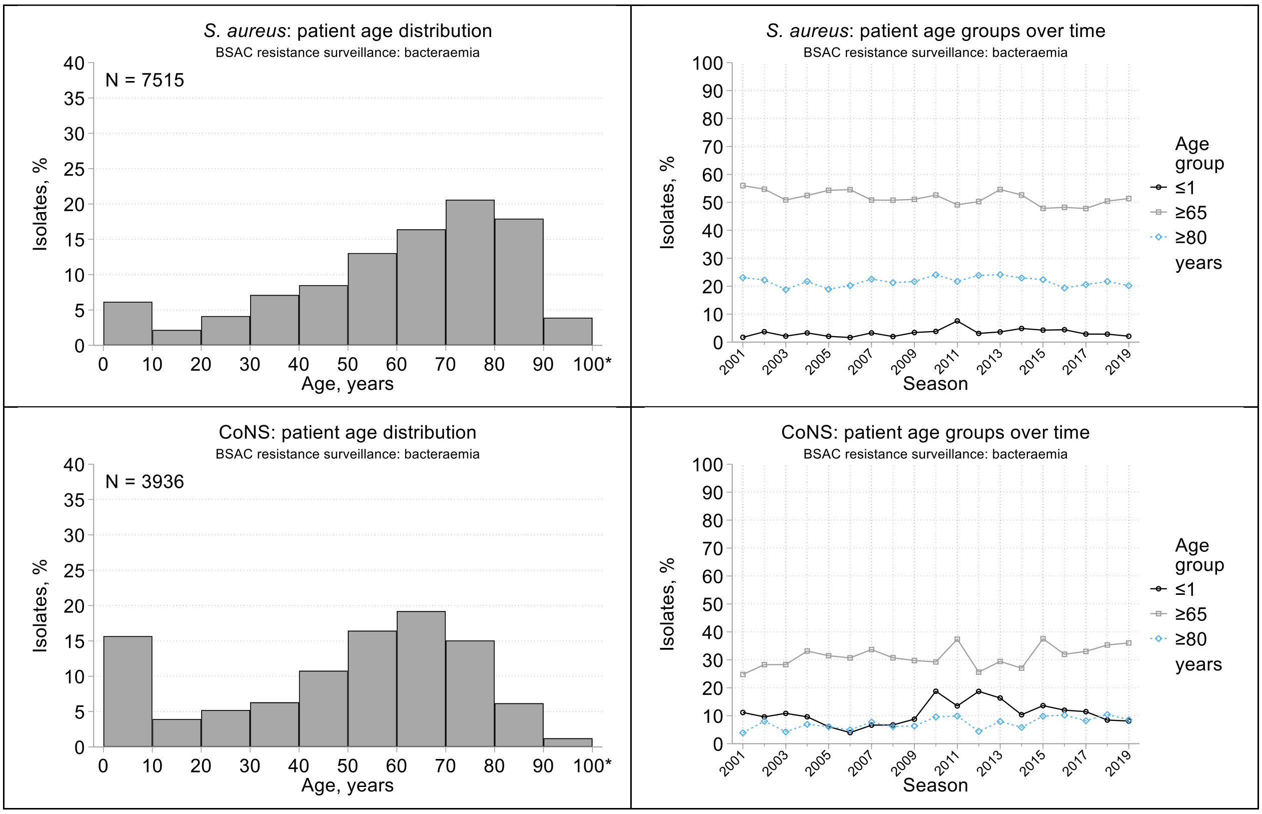

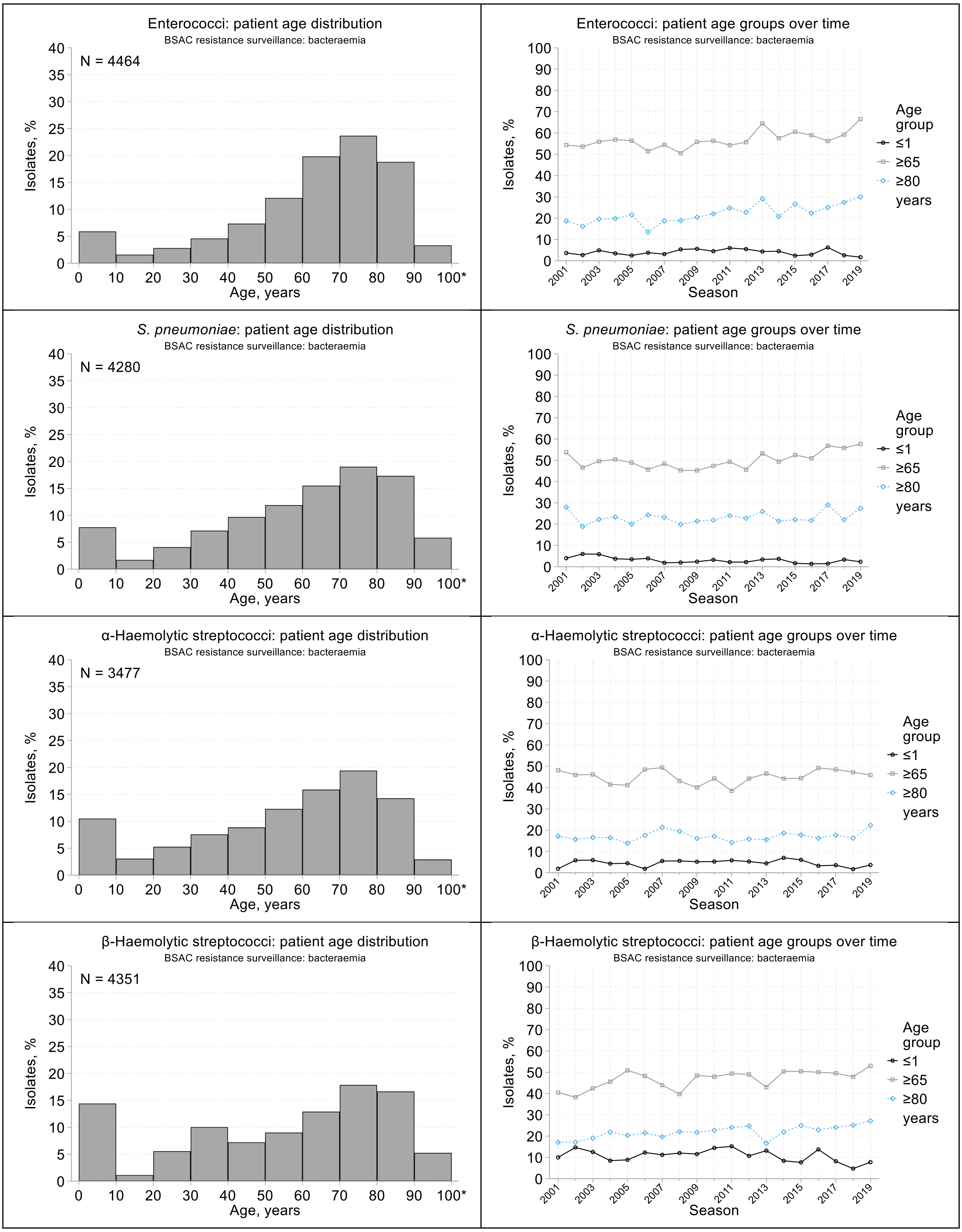

* The final bar includes all patients aged ≥90 years.

Care setting at time of sample – hospitalised >48 hours versus all other care settings (community, outpatients, hospitalised ≤48 hours).

Trends in care setting were widespread and generally consistent between organism groups, though differing in detail. The proportion of isolates taken from patients in hospital for >48 hours fell substantially for most groups, but less so for enterococci and CoNS. The proportion in hospital for ≤48 hours increased, particularly in the later part of the surveillance period; the proportion from community and outpatient settings increased to a peak around 2012 before dropping back. Care setting data was seldom missing from 2003 onwards (0.4%), but more often (9.8%) absent in 2001–02, before improvements to the data collection forms.

Figure S2. Trends in care settings by organism group

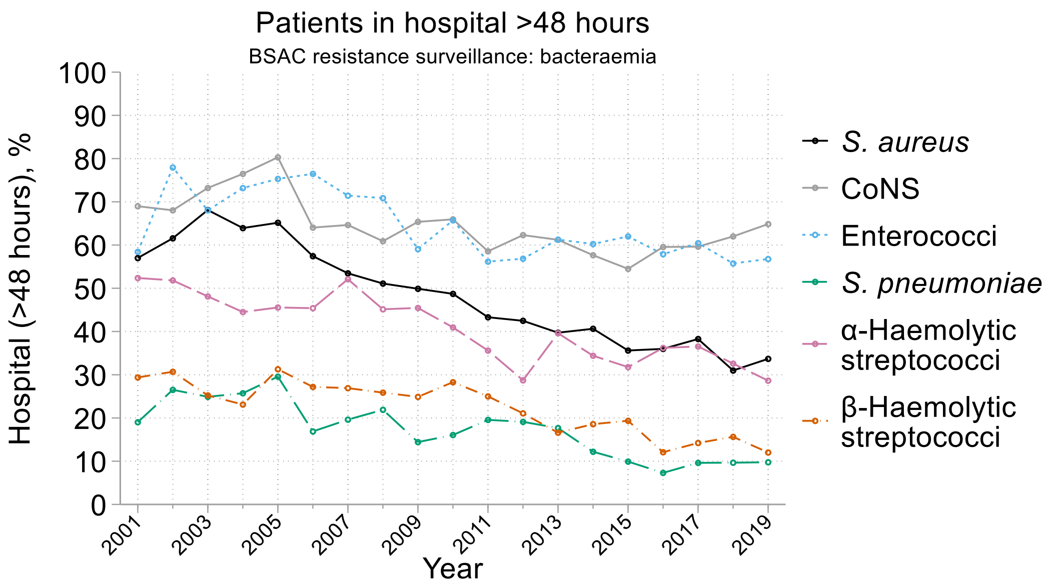

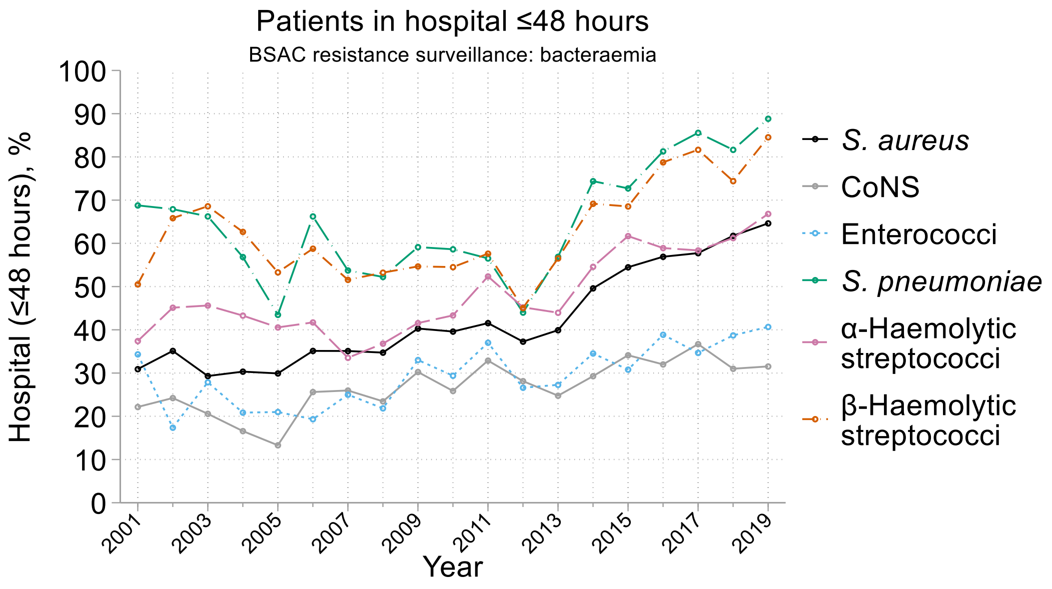

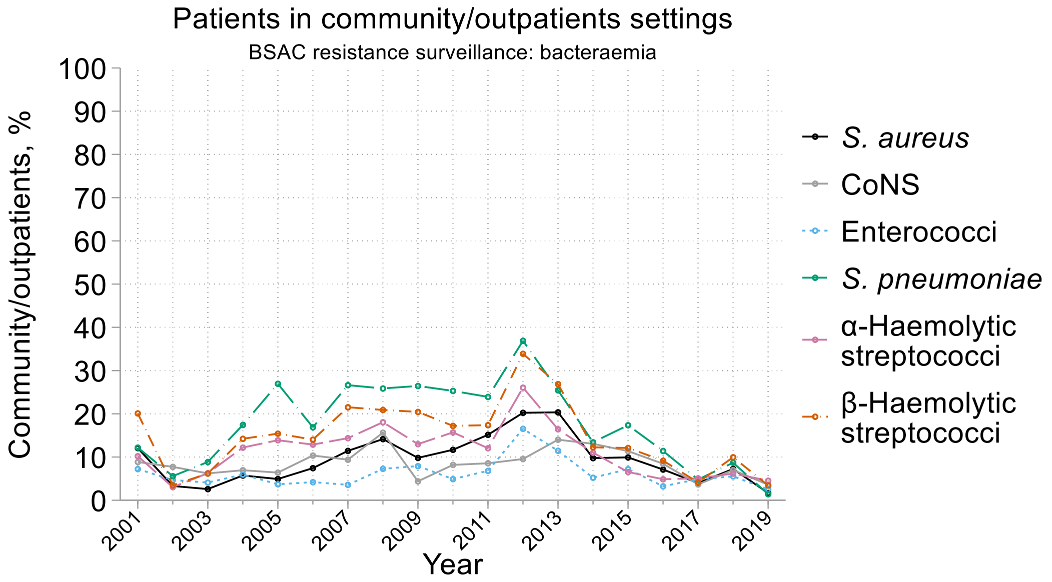

Note in all three cases above (hospital >48 hours, hospital ≤48 hours, community/outpatient settings) that the trends for *S. aureus* are not related to the falling proportion of MRSA: both MRSA and MSSA show similar patterns, as seen below.

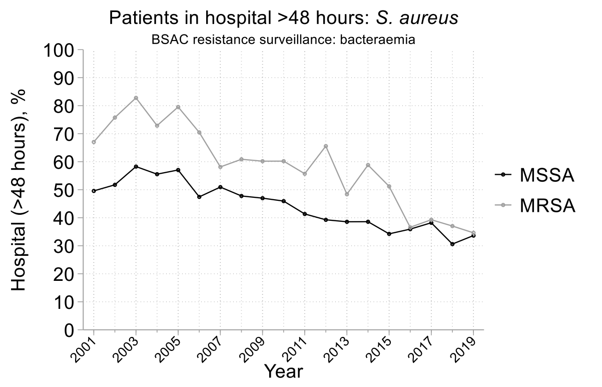

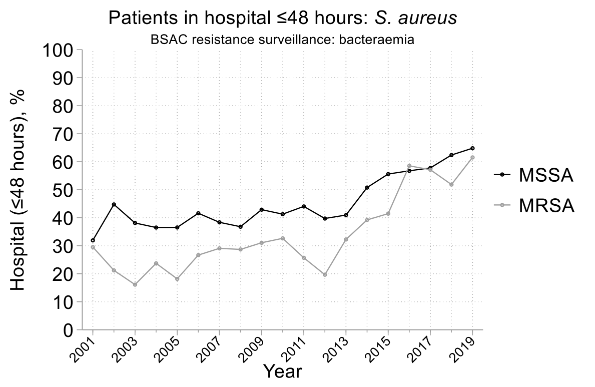

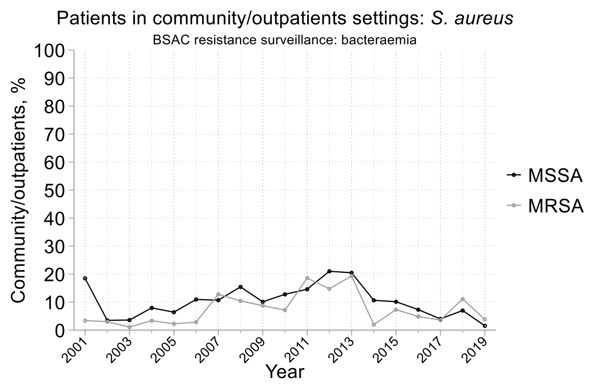

Caution: small numbers of MRSA isolates: 86–118 in 2001–10; 26–70 in 2011–19.

Care setting: intensive/critical care speciality (ICU)

Hospital speciality data from 2001–02 were discrepant and are excluded: data collection forms were improved from 2003 onwards after noting issues with recording in the 2001 data.

Table S13. Proportion of isolates from ICU patients, by organism group

Gram-positive		
Organism group	N[1]	ICU, %
S. aureus	6803	7.7
CoNS	3424	15.2
Enterococci	3894	15.4
S. pneumoniae	3729	4.7
α-Haemolytic streptococci	3024	5.0
β-Haemolytic streptococci	3823	4.9

[1] Number of isolates with speciality data, after excluding 2001–02. (Missing data: 926/25,623 = 3.6%; varied slightly between organisms from 3.2% for *S. pneumoniae* to 4.3% for α-haemolytic streptococci.)
Caveat: These tabulated 'overall' figures do not represent any particular year and may have changed over time.

Figure S3. Trends in the proportion of isolates from ICU patients by organism group

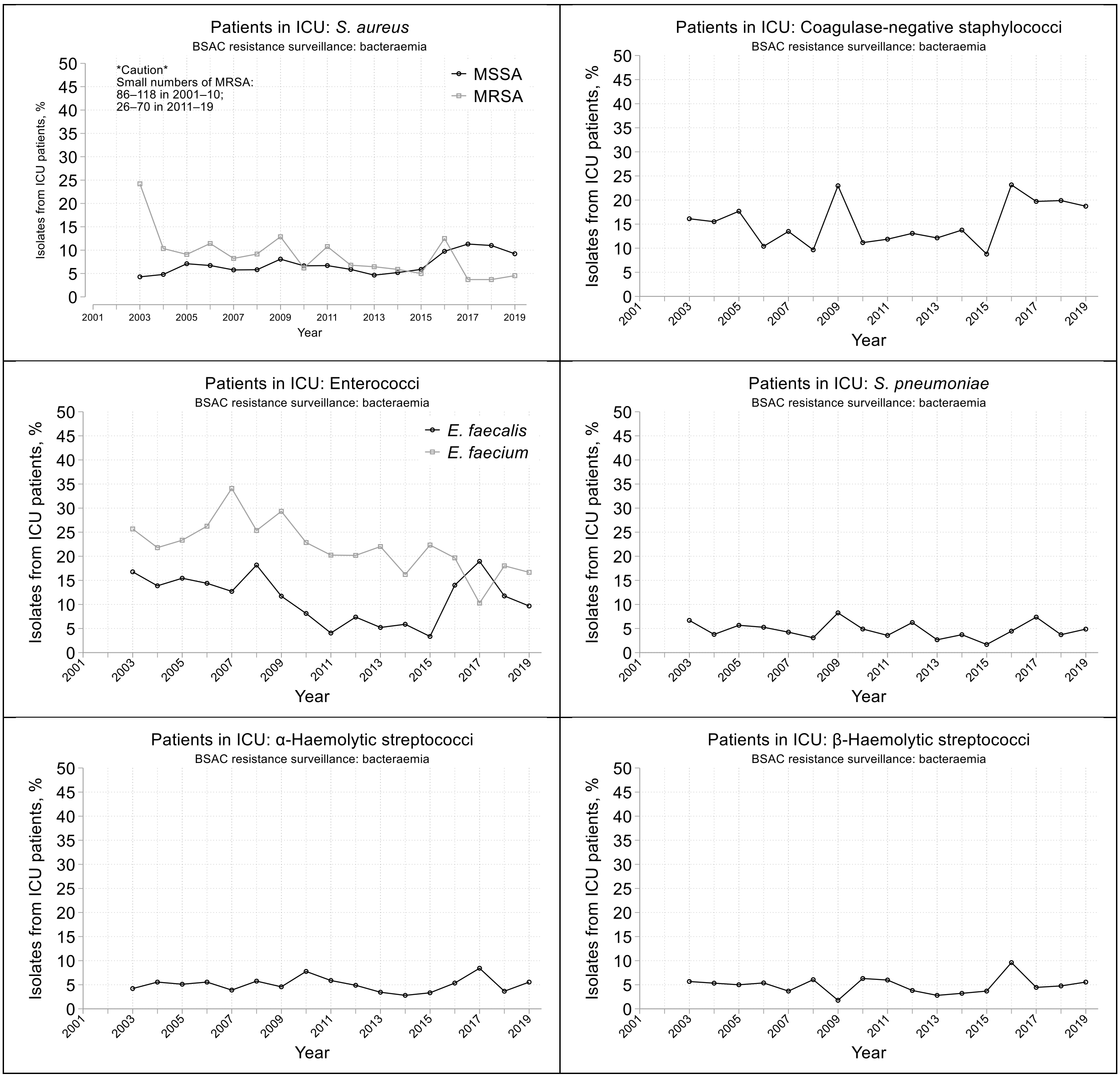

*Caution: small numbers of MRSA isolates (86–118 in 2001–10; 26–51 in 2014–19)

Source of Infection (2001–13)

From 2001–13, foci of infection and putative sources of bacteraemia were recorded as: genitourinary tract, lines, respiratory tract, gastrointestinal tract, skin and soft tissue, endocarditis, surgical site, cerebrospinal fluid, or other. However, the data quality was poor, with 4% listed as 'other' and 38% missing. In 2014–15, free text entries were accepted but were too disparate for meaningful analysis, and data collection was abandoned from 2016 onwards.

Tables S14. Source of infection: top three or all ≥10% of isolates, by organism

Organism group	N[1]	Major sources of bacteraemia
S. aureus	2798	invasive device (31%), skin / soft tissue (27%), respiratory (12%)
CoNS	1788	invasive device (83%), endocarditis (5%), other (3%)
Enterococci	1891	invasive device (31%), gastrointestinal (27%), genitourinary (20%)
S. pneumoniae	2065	respiratory (89%), cerebrospinal fluid (4%), other (4%)
α-Haemolytic streptococci	1273	endocarditis (32%), gastrointestinal (23%), invasive device (15%), respiratory (10%)
β-Haemolytic streptococci	1673	skin / soft tissue (49%), respiratory (13%), other (13%), genitourinary (10%)

[1] Number of isolates 2001–13, with data for presumed source of bacteraemia.
Caution: 38% missing data; recording may have been of poor quality.

UKHSA bacteraemia surveillance – routine data, England only

Table S15. Number of reported bacteraemias due to Gram-positive bacteria (UKHSA surveillance, routine data)

Year	Staphylococci		Enterococci	*Streptococcus*		
	S. aureus	Coagulase-negative		*S. pneumoniae*	α-haemolytic	β-haemolytic
2001	12403	5402	3673	3583	1857	2565
2002	12574	6221	4376	3888	2051	2838
2003	14112	8219	5504	4744	2539	3915
2004	13698	9553	5758	4422	2684	3573
2005	13553	11087	6337	4888	2838	3260
2006	13150	15370	7086	4487	3096	3436
2007	12111	20610	6891	4326	3493	3528
2008	10633	17049	5660	4237	3557	3901
2009	9170	16729	5393	4012	3542	4088
2010	8678	15098	5006	4116	3621	4409
2011	8111	14220	5074	3679	3671	4279
2012	7788	14786	5154	3474	3851	4447
2013	8121	14902	5221	3183	4104	4861
2014	8513	17032	5407	3219	4111	4797
2015	9483	23403	6044	4238	5109	6007
2016	10382	28808	6860	4845	5987	6824
2017	11169	36319	7089	4841	6715	7238
2018	11434	40741	7322	5139	7290	8409
2019	12068	41875	7793	5100	7783	8104
2020	11448	45093	8202	2245	6568	6838
2021	12183	52548	9232	2291	6916	5981
2022	12878	55503	9040	3632	7343	7943
2023	13190	57304	9282	4502	8055	9009
2024	13553	59335	9343	5131	8673	8268

Counts of bacteraemia episodes for 2020–2024 are presented here and in Figure S4, showing the continuing importance of the organism groups included in the BSAC surveillance, but we did not analyse them for antimicrobial resistance as we could not compare with BSAC surveillance after 2019.

Table S16. Staphylococcal bacteraemias, by methicillin status (UKHSA surveillance, routine data)

Year	*S. aureus*			coagulase-negative staphylococci		
	MSSA	MRSA	not tested	MSCoNS	MRCoNS	not tested
2001	6490	4576	1337	669	989	3744
2002	6644	4873	1057	1122	2169	2930
2003	7631	5240	1241	1959	3475	2785
2004	7719	4994	985	2421	4343	2789
2005	7738	4968	847	3069	4945	3073
2006	7816	4718	616	4586	6939	3845
2007	8199	3404	508	6307	8817	5486
2008	7806	2278	549	5054	7292	4703
2009	6749	1475	946	4444	6778	5507
2010	6655	1122	901	4077	6494	4527
2011	6520	867	724	4043	6648	3529
2012	6287	826	675	4168	6907	3711
2013	6652	780	689	4251	7121	3530
2014	6502	780	1231	4189	7742	5101
2015	7218	662	1603	6005	10161	7237
2016	7790	588	2004	7489	11689	9630
2017	8522	720	1927	9637	13871	12811
2018	9377	818	1239	11640	15235	13866
2019	9797	817	1454	12210	14817	14848

Methicillin resistance was inferred if reported resistant to any one or more of oxacillin, cloxacillin or cefoxitin.

Figure S4. Numbers of reported Gram-positive bacteraemias with organisms included in BSAC surveillance (UKHSA surveillance, routine data).

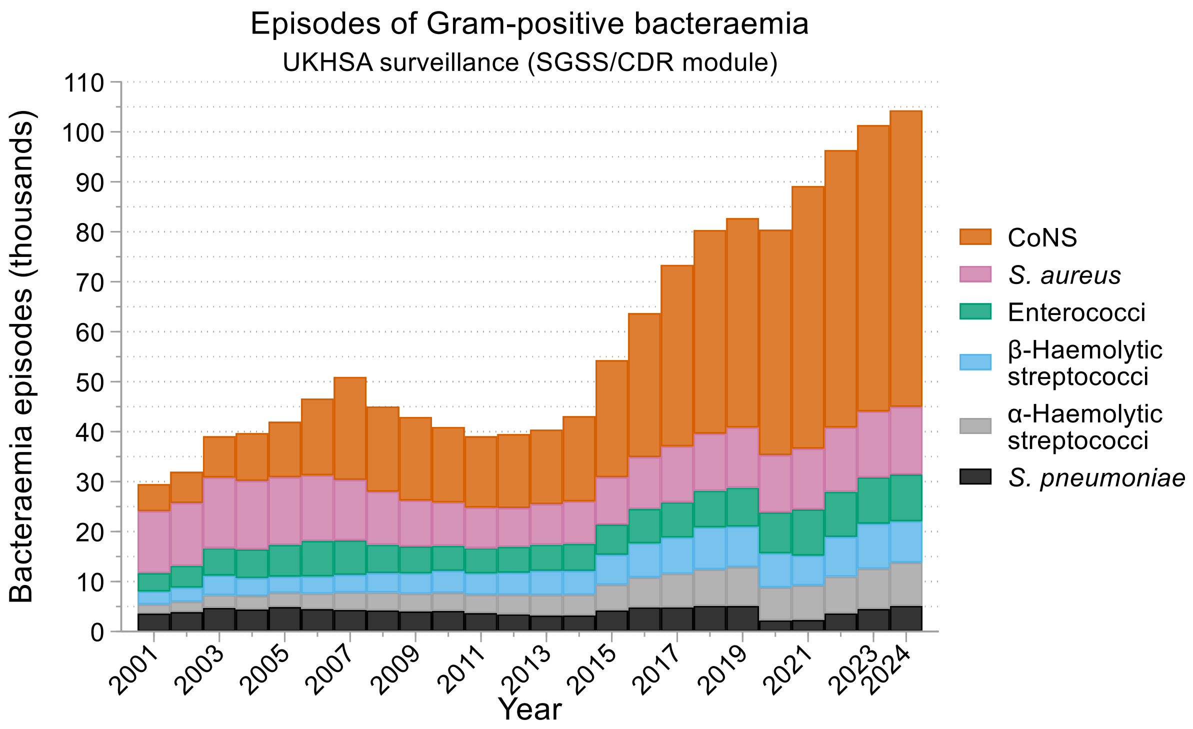

Figure S5 Trends in percentage of bacteraemias reported to UKHSA with antimicrobial susceptibility test results, by organism group (UKHSA surveillance, routine data)

In general, reporting of antibiotic susceptibility testing results increased over time, reaching a plateau around 90% for well-established antimicrobial agents. Figures 5.2b and 5.2c show an unusual pattern for vancomycin testing in CoNS, with reported testing falling rapidly from around 90% in 2010 to approximately 30% for MSCoNS and 40% for MRCoNS by 2019. There was a smaller fall in vancomycin testing for *S. aureus* over the same period. The trends for testing teicoplanin in this time were contradictory – continuing to rise for *S. aureus* but falling for CoNS.

KEY to antimicrobials: resistance data present for ...

AMP: ampicillin
DAP: daptomycin
ERY: erythromycin
FUS: fusidic acid
LZD: linezolid
PEN: penicillin
RIF: rifampicin
TEC: teicoplanin
TET: tetracycline
TGC: tigecycline
VAN: vancomycin

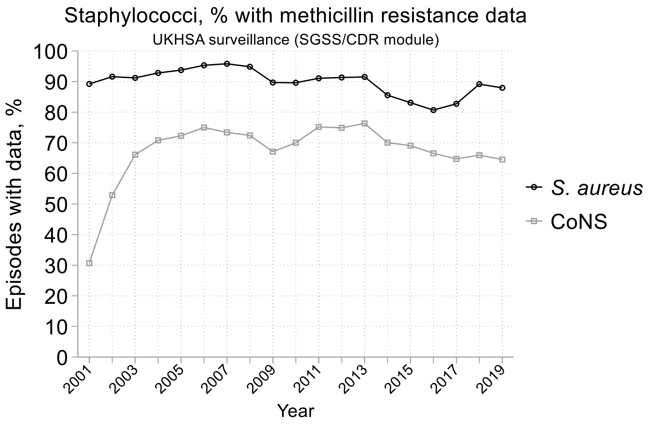

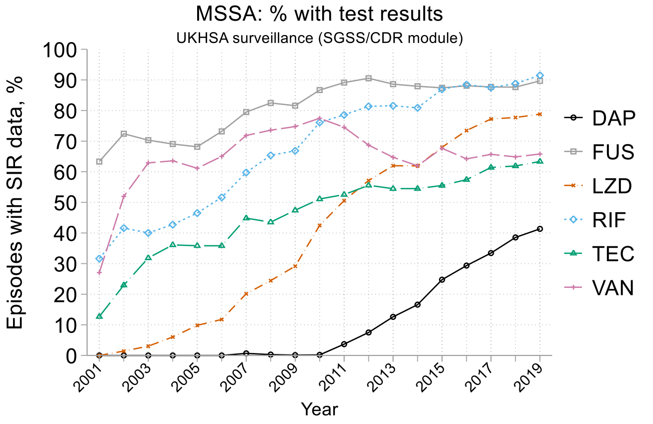

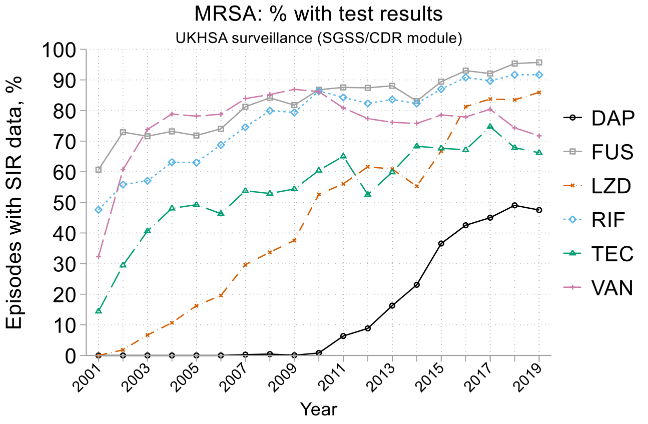

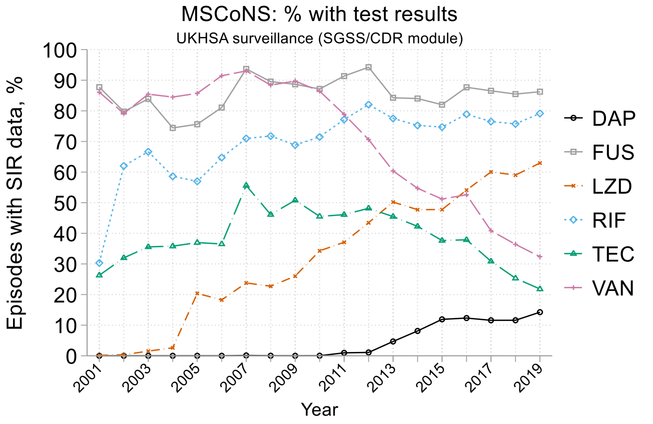

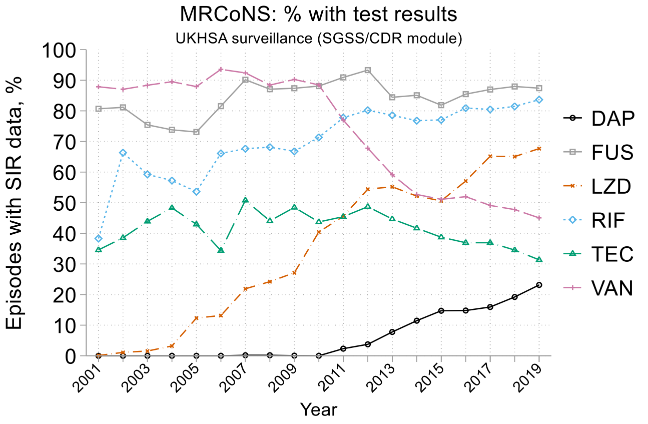

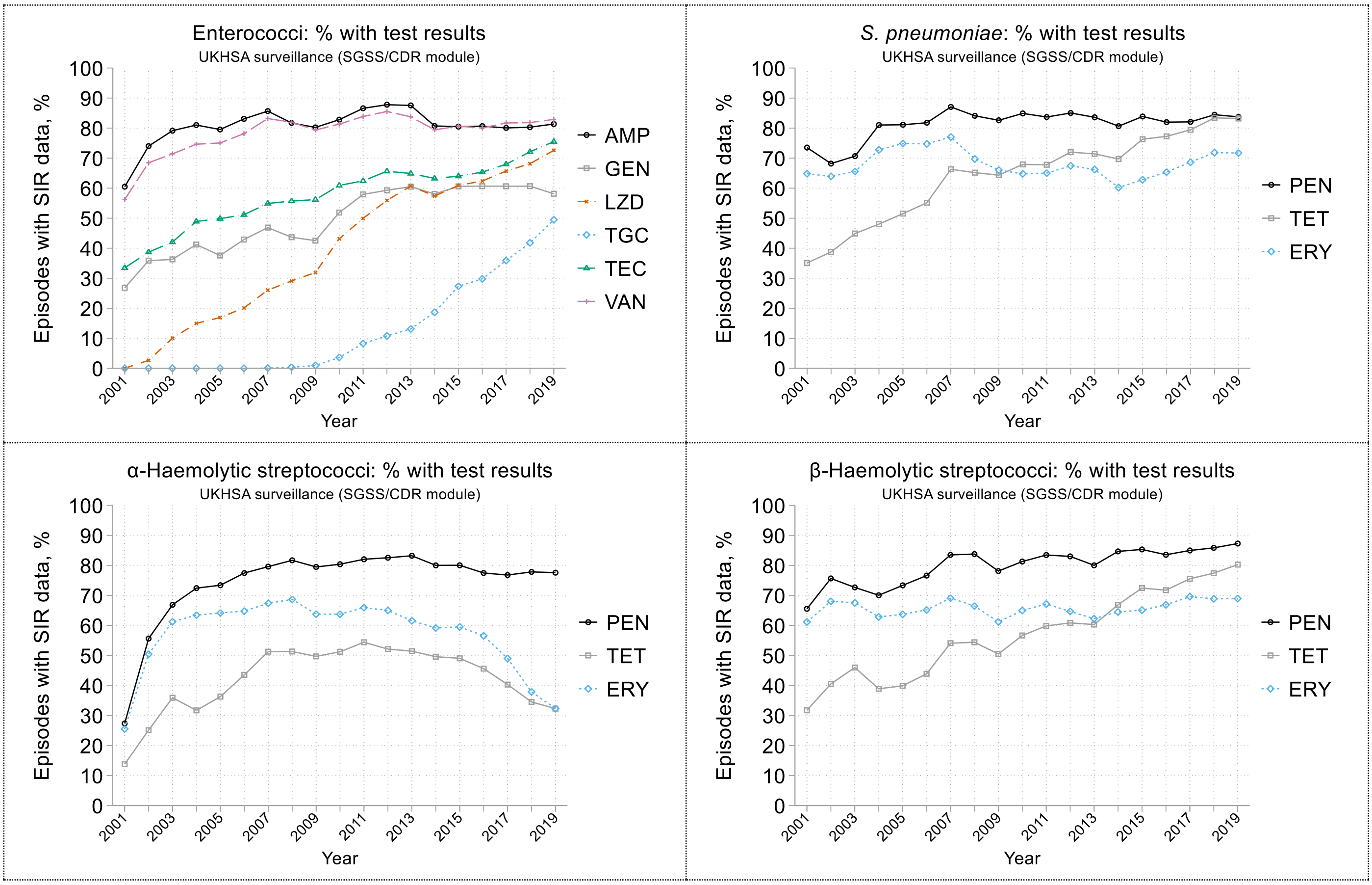

Enterococci: % with test results
UKHSA surveillance (SGSS/CDR module)
Episodes with SIR data, %
Year
AMP
GEN
LZD
TGC
TEC
VAN
S. pneumoniae: % with test results
UKHSA surveillance (SGSS/CDR module)
Episodes with SIR data, %
Year
PEN
TET
ERY
α-Haemolytic streptococci: % with test results
UKHSA surveillance (SGSS/CDR module)
Episodes with SIR data, %
Year
PEN
TET
ERY
β-Haemolytic streptococci: % with test results
UKHSA surveillance (SGSS/CDR module)
Episodes with SIR data, %
Year
PEN
TET
ERY

References

1. Allen M, Reynolds R, Mushtaq S *et al*. The British Society for Antimicrobial Chemotherapy Resistance Surveillance Project: methods and limitations. *J Antimicrob Chemother* 2025; **80** (Suppl 4): iv7–iv21.

2. Reacher MH, Shah A, Livermore DM *et al*. Bacteraemia and antibiotic resistance of its pathogens reported in England and Wales between 1990 and 1998: trend analysis. *BMJ* 2000; **320:** 213–6.

3. Wilson J, Elgohari S, Livermore DM *et al*. Trends among pathogens reported as causing bacteraemia in England, 2004-2008. *Clin Microbiol Infect* 2011; **17:** 451–8.

4. eucast: Clinical breakpoints and dosing of antibiotics. Available from: https://www.eucast.org/clinical_breakpoints/

5. Scottish Antimicrobial Prescribing Group. Antimicrobial-specific guidance. Available from: https://www.sapg.scot/guidance-qi-tools/antimicrobial-specific-guidance/

APPENDIX – MIC distributions

BSAC Gram-positive bacteraemia resistance surveillance

These graphs are presented as thumbnails for reasons of space. Please zoom in to read.
The red vertical lines show EUCAST v12.0 (2022) breakpoint(s)(4) or, if used for analysis in the absence of breakpoints, ECOFFs – see Tables S5–S10. Where two lines are shown, they indicate the susceptible (S ≤) and resistant (R >) breakpoints; MICs between these bounds are designated I “susceptible, increased exposure”. More commonly, there is a single line because the S and R breakpoints are coincident and there is no I category. Occasionally, noted below, there is no S category, and the single line demarcates R from I.

Some distributions were affected by excessive censoring due to testing of restricted concentration ranges, usually in earlier years. These years’ data are omitted, as noted in the affected plots, to show the true range more accurately.

Collection years and number of isolates are noted within each plot. With few exceptions,* MIC axes all span ≤0.001 to ≥1024 mg/L, with labelled values showing the range of MICs actually observed in those years.

Combinations lacking S category
Staphylococci: ciprofloxacin
Enterococci: gentamicin
α-haemolytic streptococci: gentamicin

** Exceptional extensions of MIC (x-) axis:*
Enterococci/gentamicin: to ≥4096 mg/L)
Staphylococci/mupirocin: to ≥2048

Staphylococci

S. aureus

Coagulase-negative staphylococci

MSSA

Ceftaroline

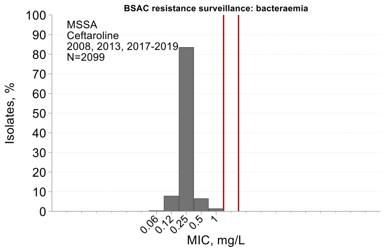

MRSA

Ceftaroline

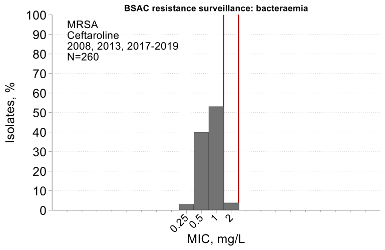

MSCoNS

Ceftaroline

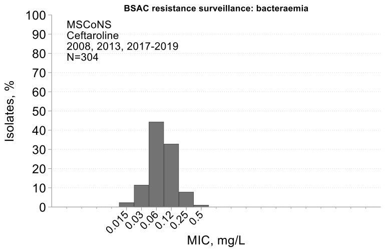

MRCoNS

Ceftaroline

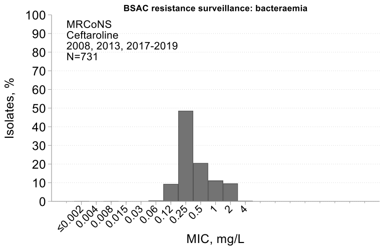

Ceftobiprole

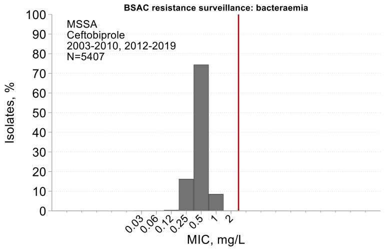

Ceftobiprole

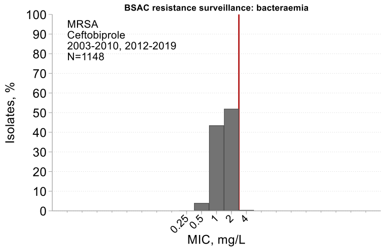

Ceftobiprole

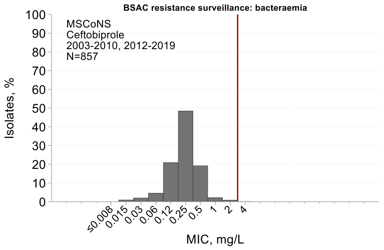

Ceftobiprole

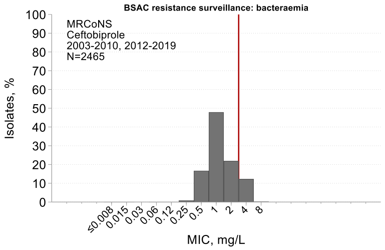

Ciprofloxacin

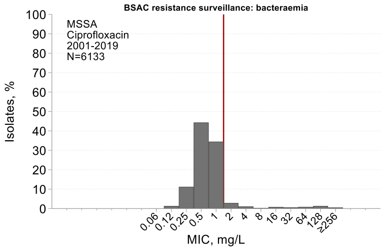

Ciprofloxacin

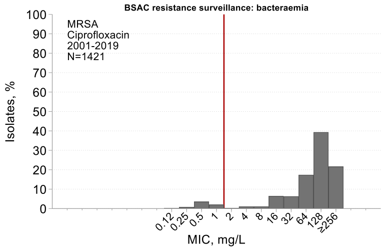

Ciprofloxacin

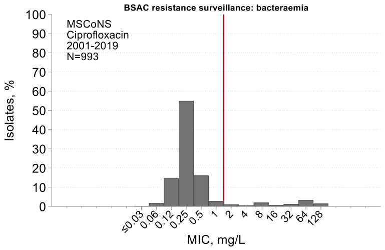

Ciprofloxacin

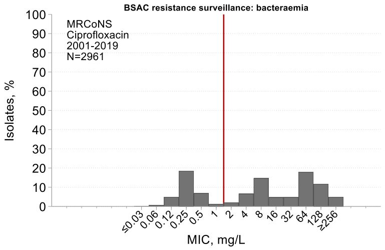

Clindamycin

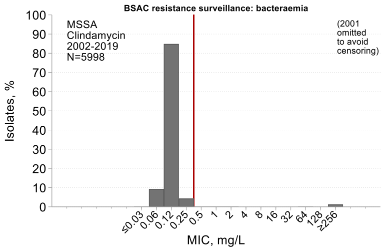

Clindamycin

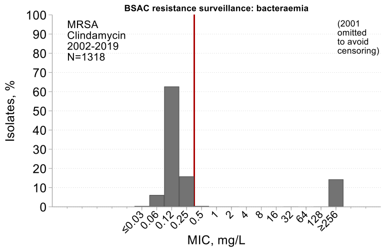

Clindamycin

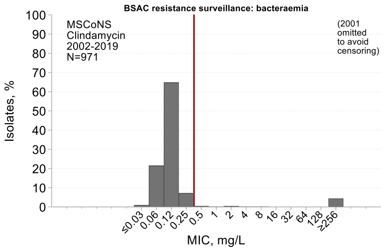

Clindamycin

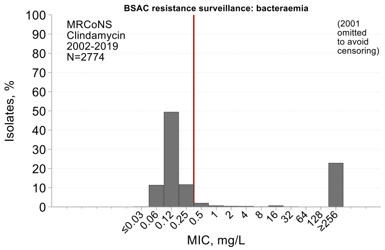

S. aureus		Coagulase-negative staphylococci	
**MSSA**	**MRSA**	**MSCoNS**	**MRCoNS**

Daptomycin

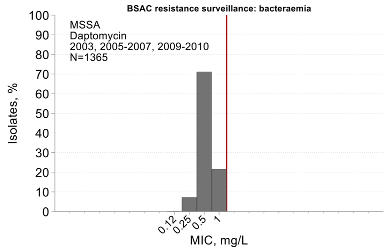

Daptomycin

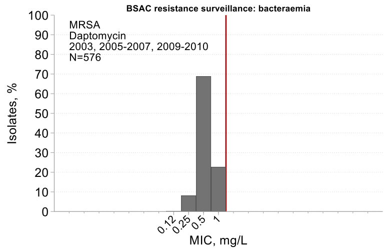

Daptomycin

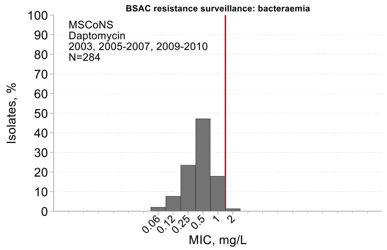

Daptomycin

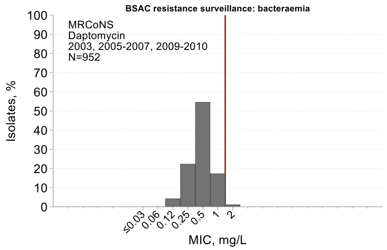

Erythromycin

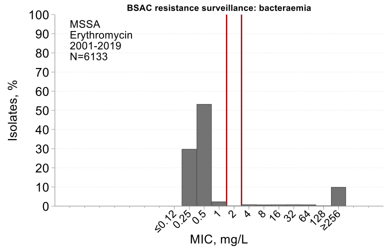

Erythromycin

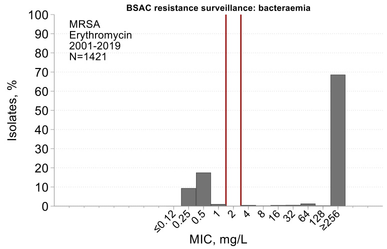

Erythromycin

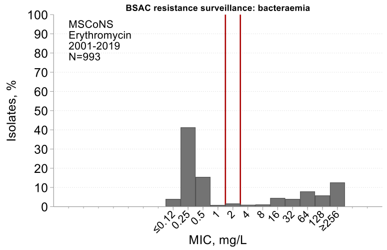

Erythromycin

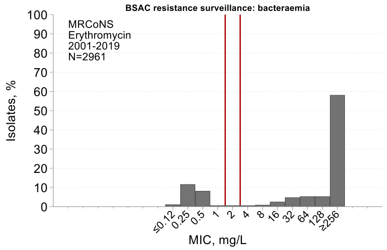

Fusidic acid

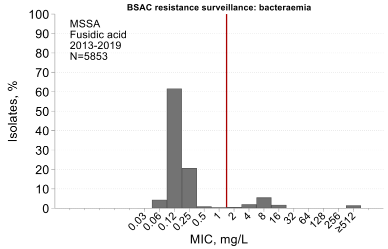

Fusidic acid

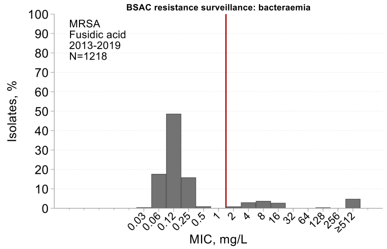

Fusidic acid

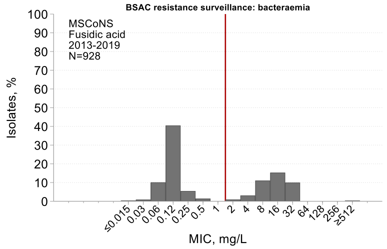

Fusidic acid

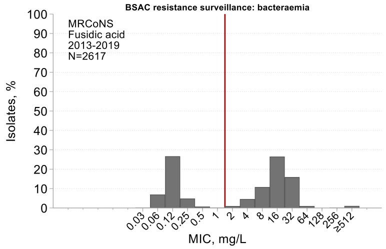

Gentamicin

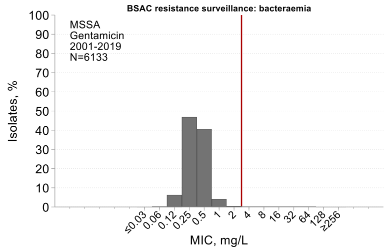

Gentamicin

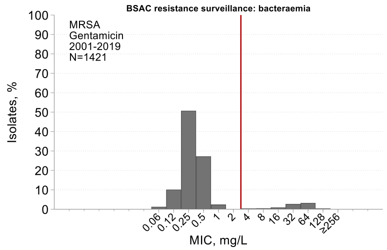

Gentamicin

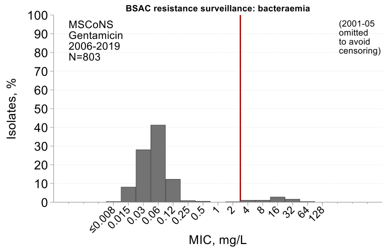

Gentamicin

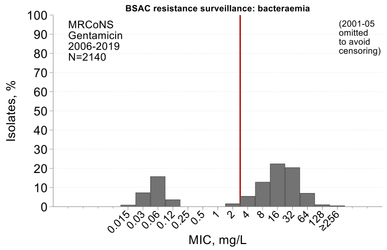

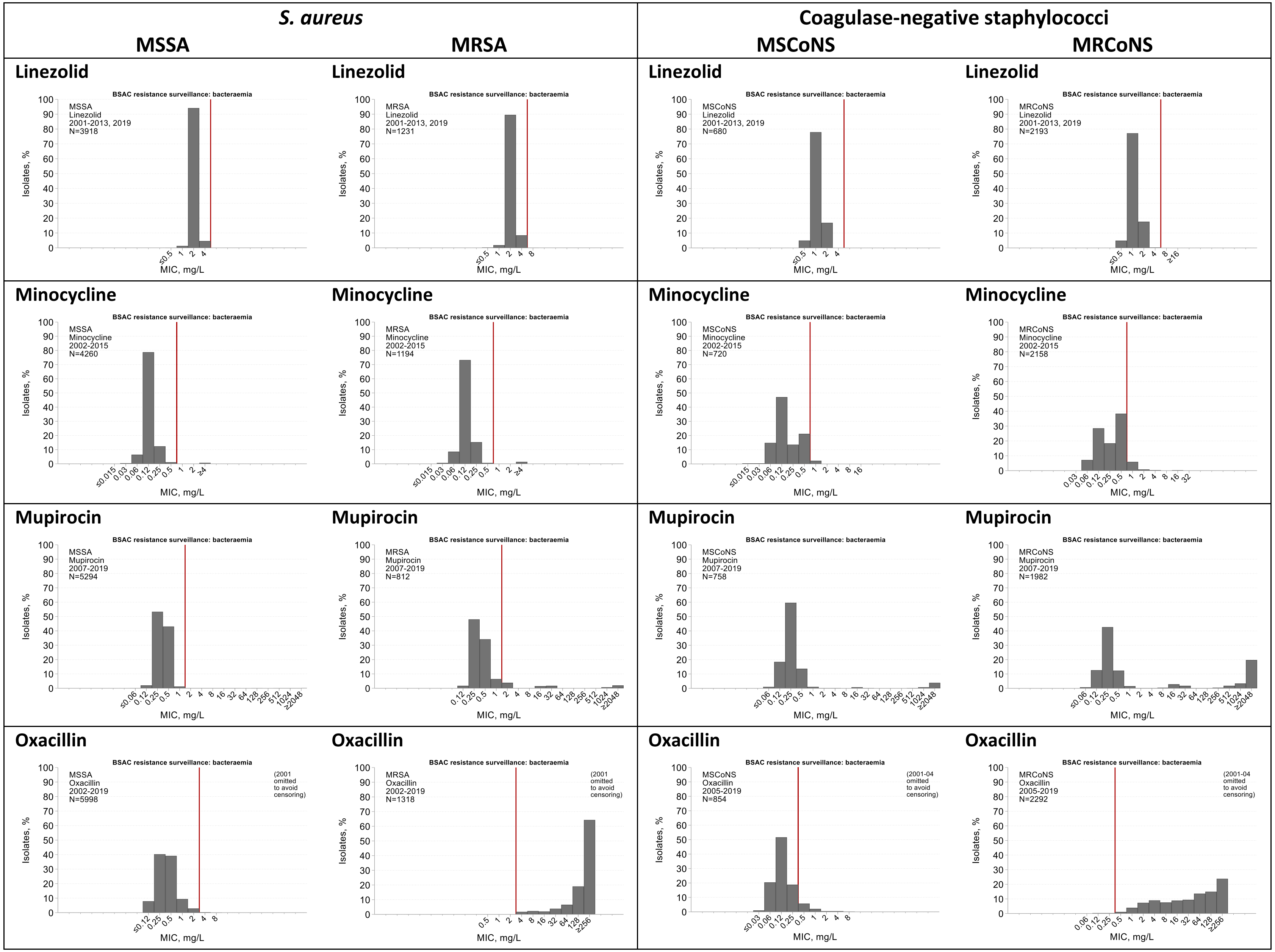
S. aureus
Coagulase-negative staphylococci
MSSA
MRSA
MSCoNS
MRCoNS
Linezolid
BSAC resistance surveillance: bacteraemia
MSSA Linezolid 2001-2013, 2019 N=3918
MRSA Linezolid 2001-2013, 2019 N=1231
MSCoNS Linezolid 2001-2013, 2019 N=680
MRCoNS Linezolid 2001-2013, 2019 N=2193
Minocycline
MSSA Minocycline 2002-2015 N=4260
MRSA Minocycline 2002-2015 N=1194
MSCoNS Minocycline 2002-2015 N=720
MRCoNS Minocycline 2002-2015 N=2158
Mupirocin
MSSA Mupirocin 2007-2019 N=5294
MRSA Mupirocin 2007-2019 N=812
MSCoNS Mupirocin 2007-2019 N=758
MRCoNS Mupirocin 2007-2019 N=1982
Oxacillin
MSSA Oxacillin 2002-2019 N=5998 (2001 omitted to avoid censoring)
MRSA Oxacillin 2002-2019 N=1318 (2001 omitted to avoid censoring)
MSCoNS Oxacillin 2005-2019 N=854 (2001-04 omitted to avoid censoring)
MRCoNS Oxacillin 2005-2019 N=2292 (2001-04 omitted to avoid censoring)
Isolates, %
MIC, mg/L

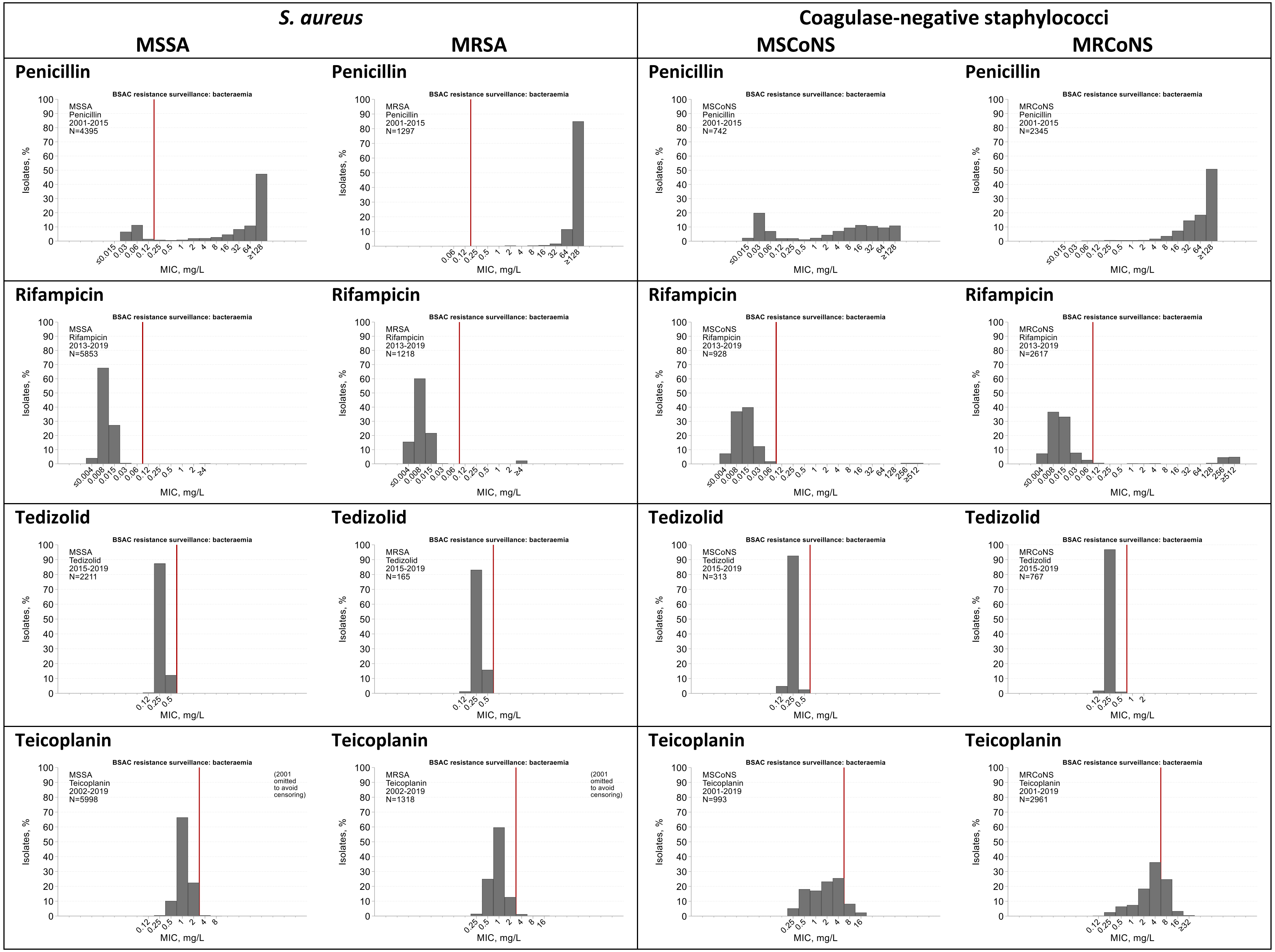
S. aureus
Coagulase-negative staphylococci
MSSA
MRSA
MSCoNS
MRCoNS
Penicillin
BSAC resistance surveillance: bacteraemia
MSSA Penicillin 2001-2015 N=4395
MRSA Penicillin 2001-2015 N=1297
MSCoNS Penicillin 2001-2015 N=742
MRCoNS Penicillin 2001-2015 N=2345
Rifampicin
MSSA Rifampicin 2013-2019 N=5853
MRSA Rifampicin 2013-2019 N=1218
MSCoNS Rifampicin 2013-2019 N=928
MRCoNS Rifampicin 2013-2019 N=2617
Tedizolid
MSSA Tedizolid 2015-2019 N=2211
MRSA Tedizolid 2015-2019 N=165
MSCoNS Tedizolid 2015-2019 N=313
MRCoNS Tedizolid 2015-2019 N=767
Teicoplanin
MSSA Teicoplanin 2002-2019 N=5998
(2001 omitted to avoid censoring)
MRSA Teicoplanin 2002-2019 N=1318
(2001 omitted to avoid censoring)
MSCoNS Teicoplanin 2001-2019 N=993
MRCoNS Teicoplanin 2001-2019 N=2961
Isolates, %
MIC, mg/L

S. aureus		Coagulase-negative staphylococci	
**MSSA**	**MRSA**	**MSCoNS**	**MRCoNS**

Tetracycline

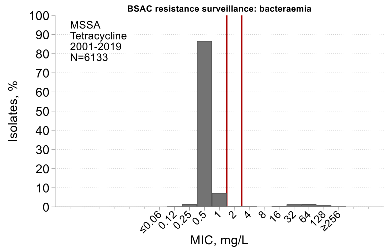

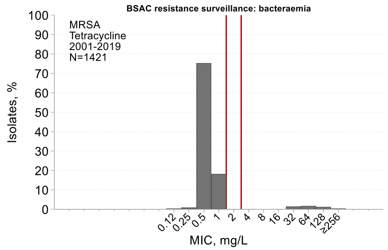

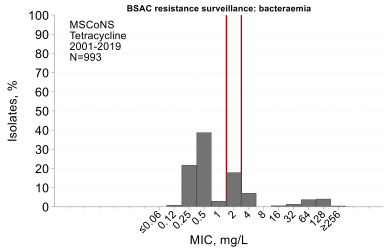

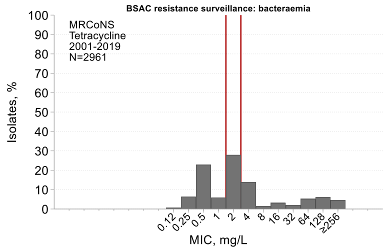

Tigecycline

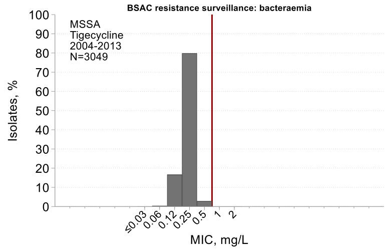

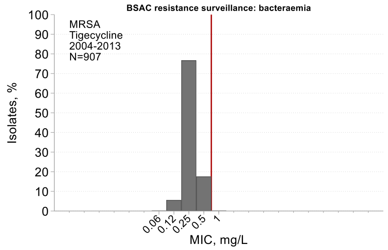

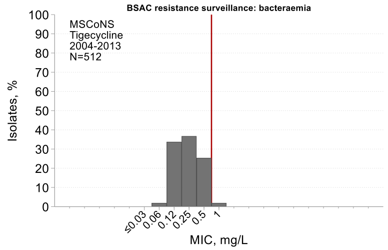

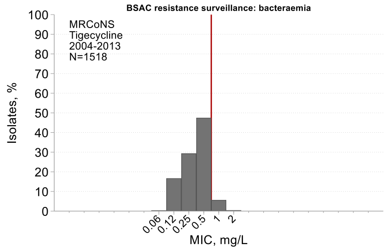

Trimethoprim

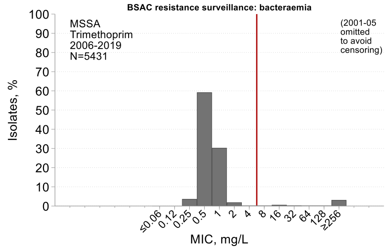

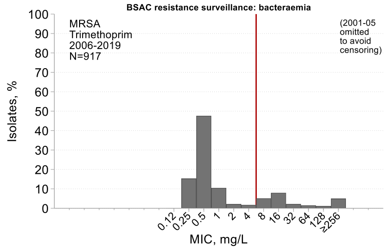

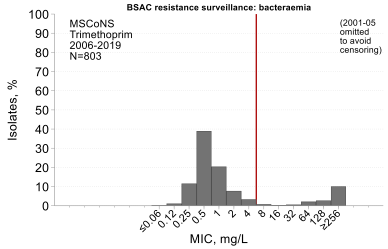

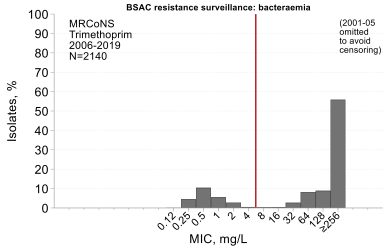

Vancomycin

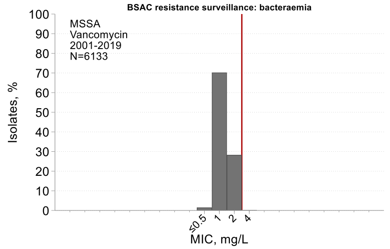

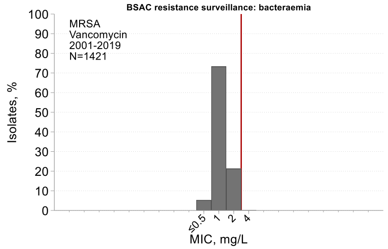

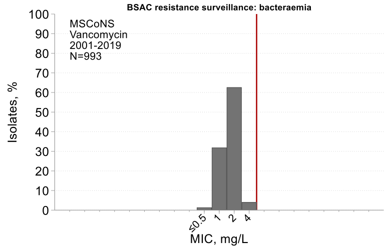

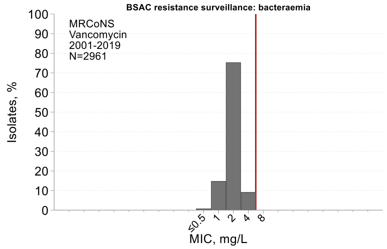

Enterococci

E. faecalis	*E. faecium*

Ampicillin

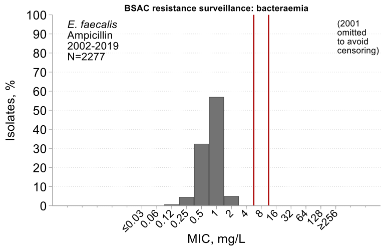

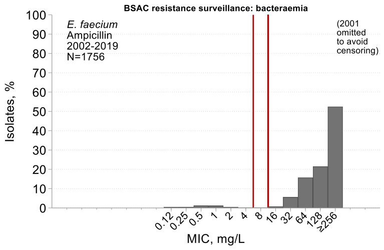

Ciprofloxacin

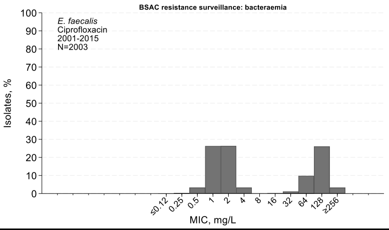

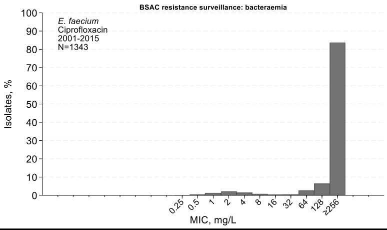

Daptomycin

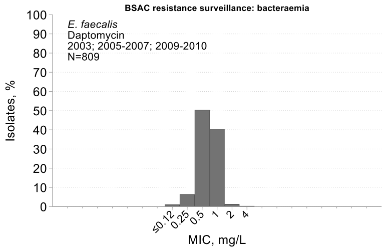

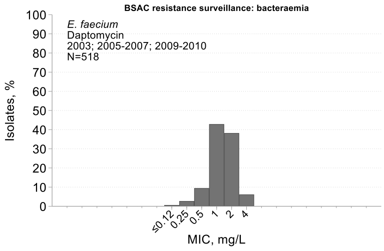

Gentamicin

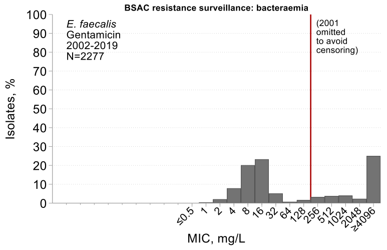

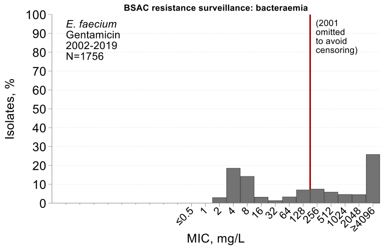

Linezolid

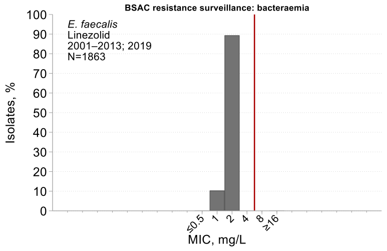

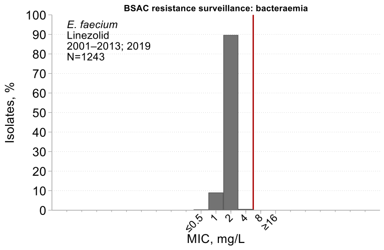

Tedizolid

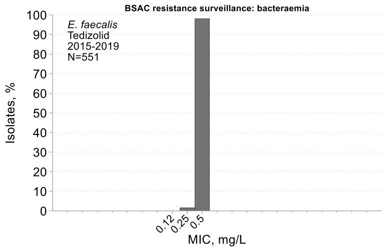

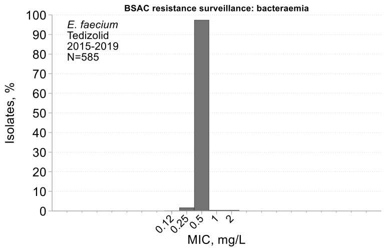

Teicoplanin

E. faecalis

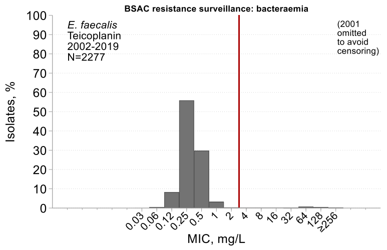

E. faecium

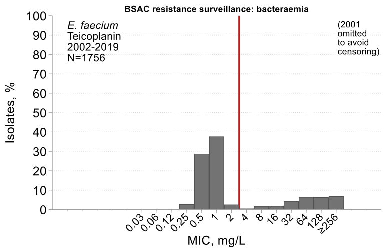

Tigecycline

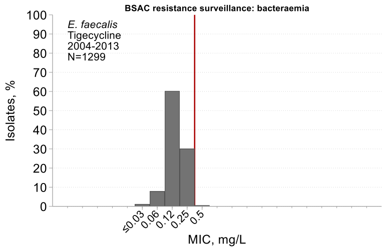

Tigecycline

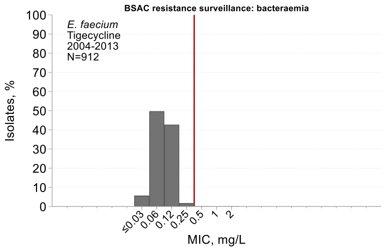

Vancomycin

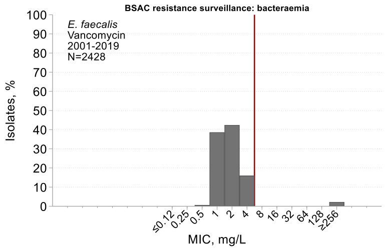

Vancomycin

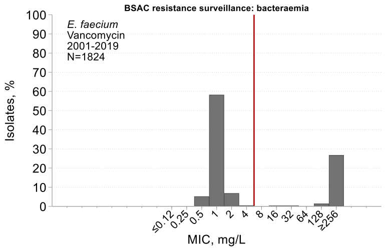

S. pneumoniae

S. pneumoniae

Amoxicillin

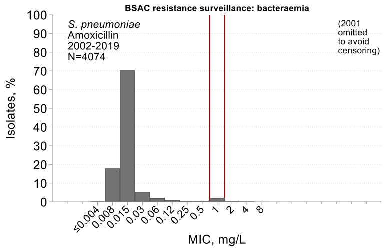

Cefotaxime

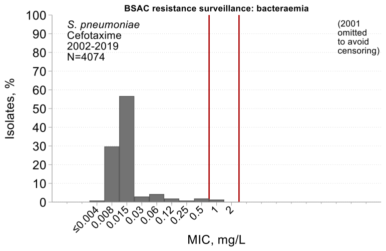

Ceftaroline

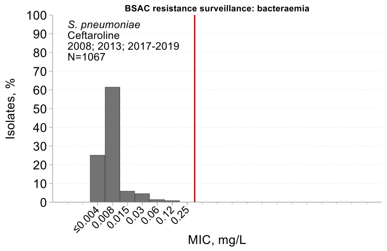

Ceftobiprole

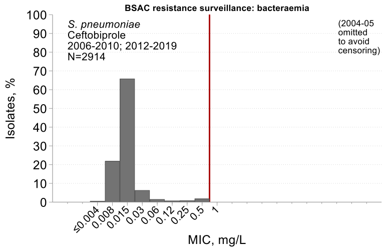

Clindamycin

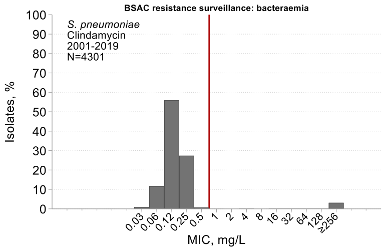

Erythromycin

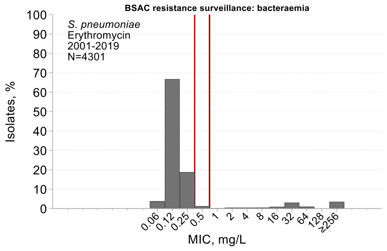

Imipenem

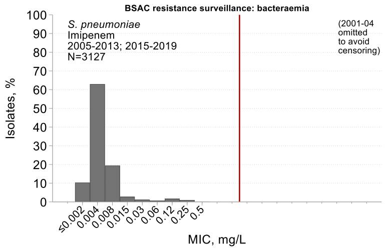

Linezolid

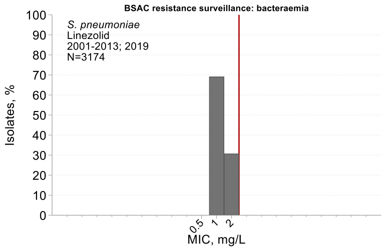

Meropenem

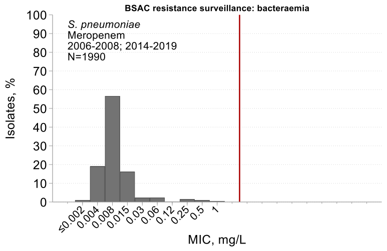

Penicillin

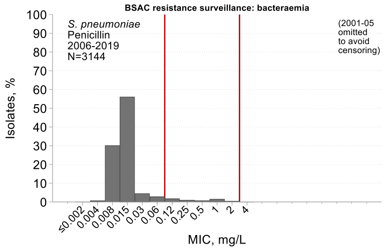

Teicoplanin

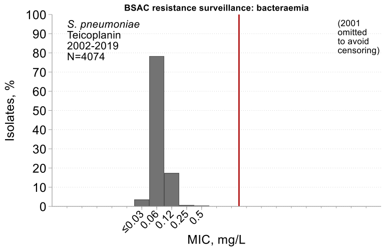

Tetracycline

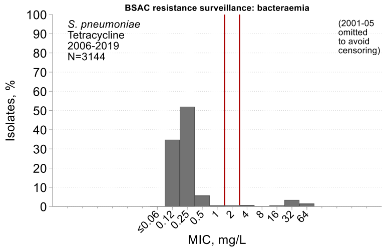

Vancomycin

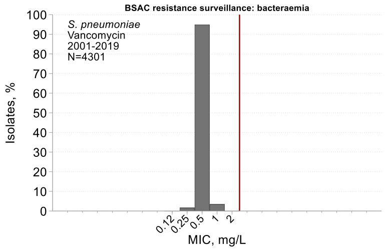

α- and non-Haemolytic streptococci

α- and non-Haemolytic streptococci

Cefotaxime

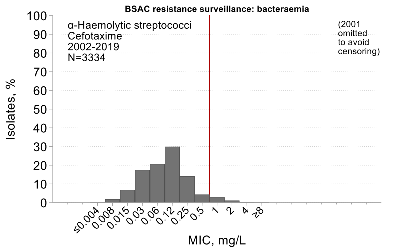

Ceftobiprole

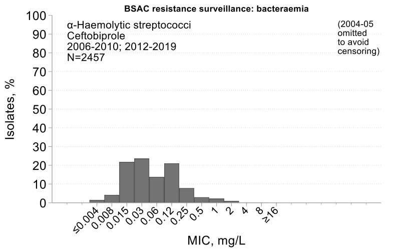

Clindamycin

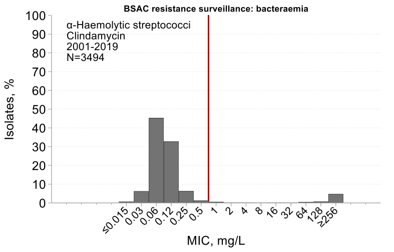

Daptomycin

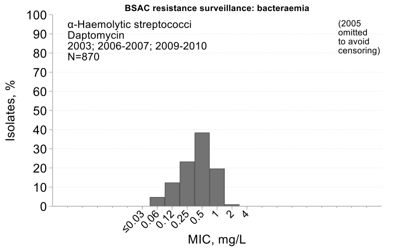

Erythromycin

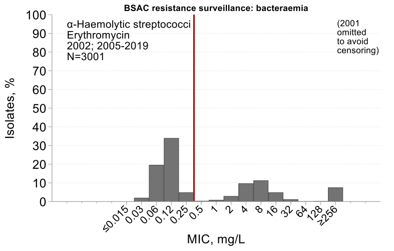

Gentamicin

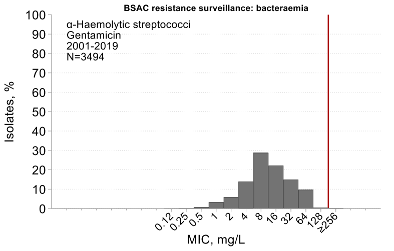

Linezolid

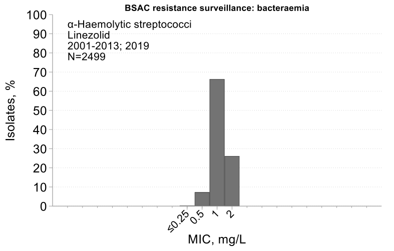

Penicillin

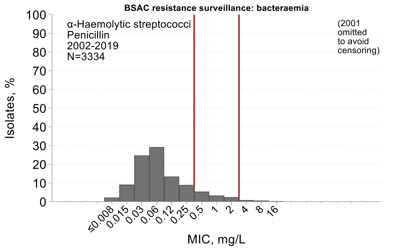

Tedizolid

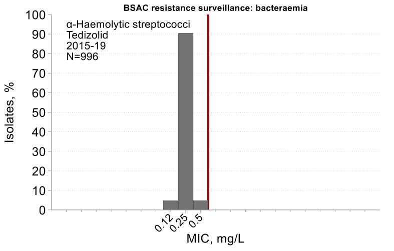

Teicoplanin

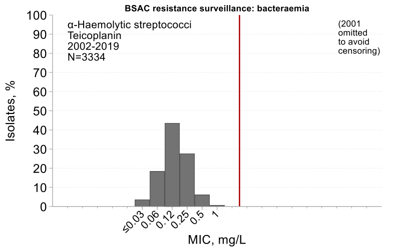

Tetracycline

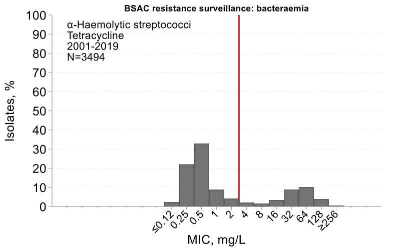

Tigecycline

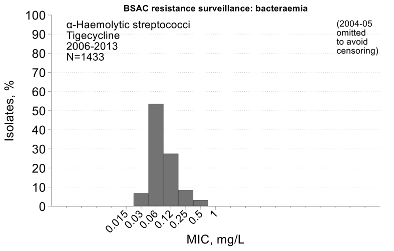

Vancomycin

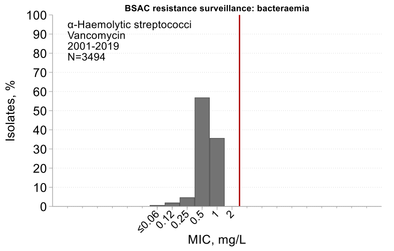

β-Haemolytic streptococci

β-Haemolytic streptococci

Group A	Group B	Group C/G

Cefotaxime (Group A)

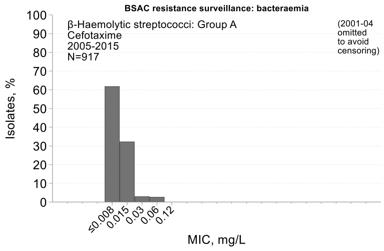

Cefotaxime (Group B)

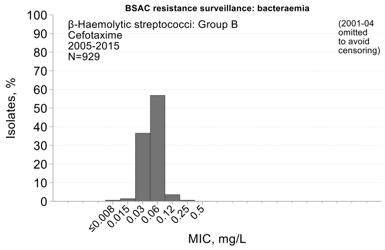

Cefotaxime (Group C/G)

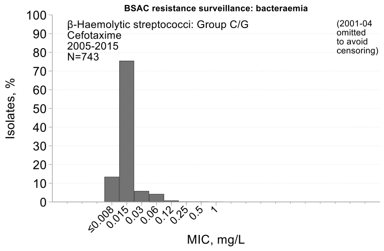

Ceftobiprole (Group A)

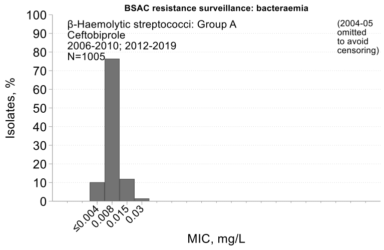

Ceftobiprole (Group B)

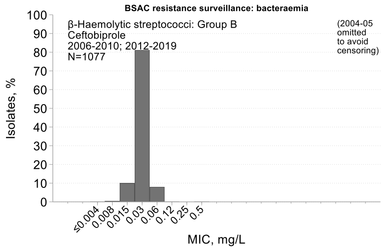

Ceftobiprole (Group C/G)

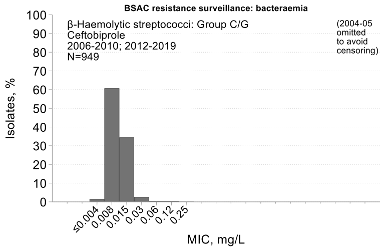

Clindamycin (Group A)

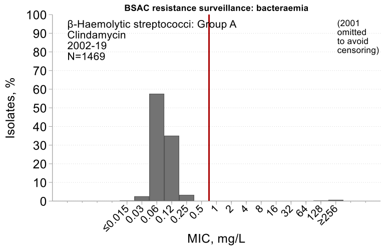

Clindamycin (Group B)

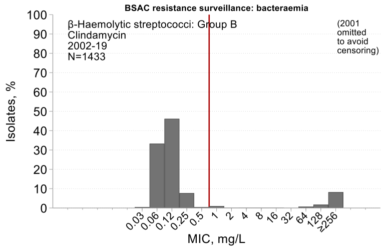

Clindamycin (Group C/G)

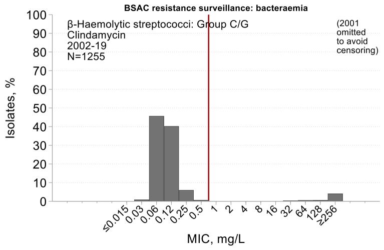

Daptomycin (Group A)

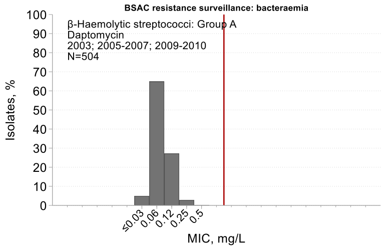

Daptomycin (Group B)

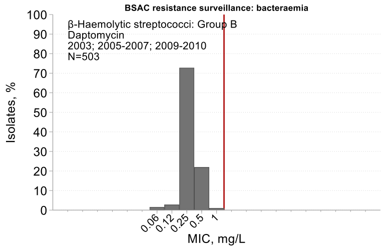

Daptomycin (Group C/G)

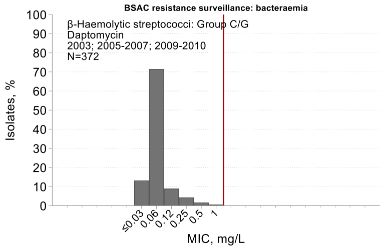

Erythromycin (Group A)

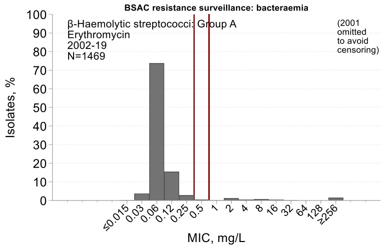

Erythromycin (Group B)

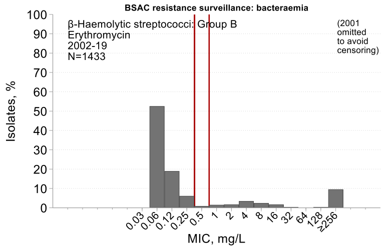

Erythromycin (Group C/G)

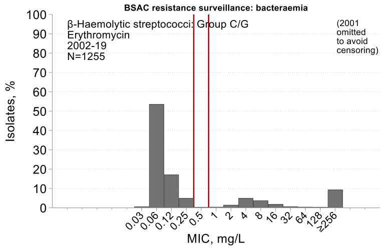

Gentamicin (Group A)

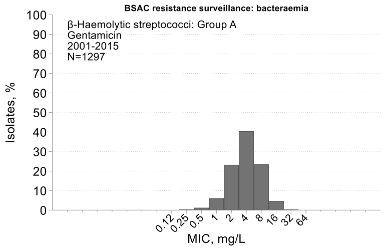

Gentamicin (Group B)

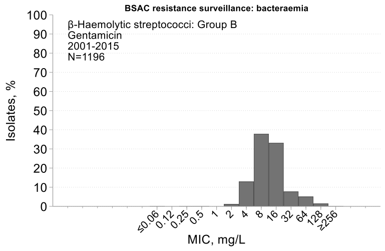

Gentamicin (Group C/G)

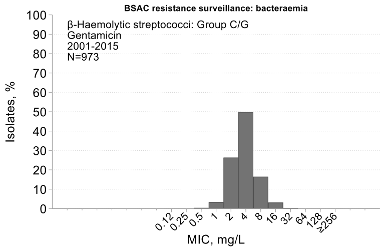

β-Haemolytic streptococci

Group A	Group B	Group C/G

Linezolid

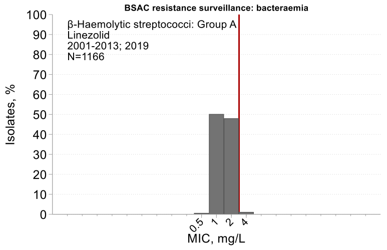

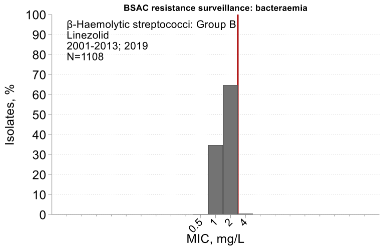

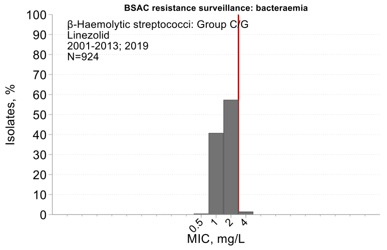

Penicillin

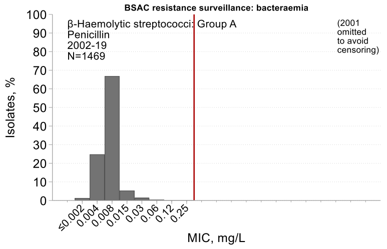

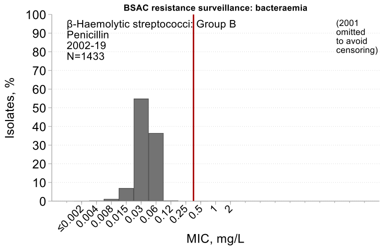

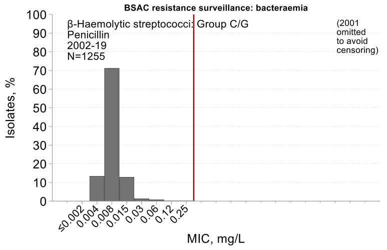

Tedizolid

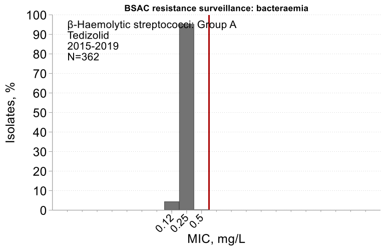

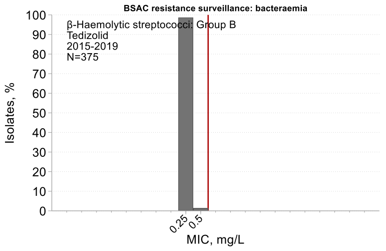

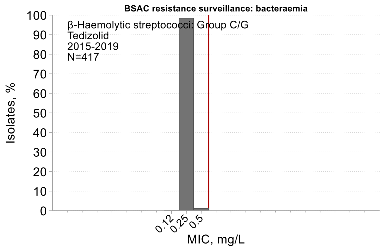

Teicoplanin

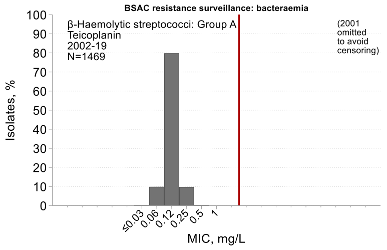

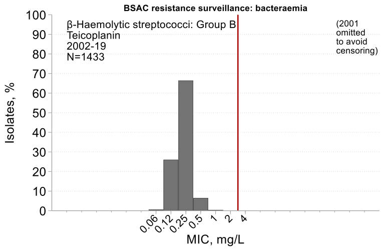

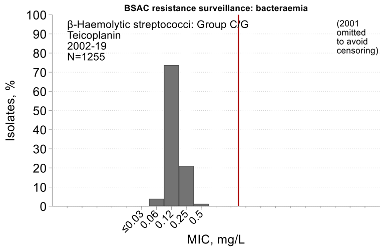

Tetracycline

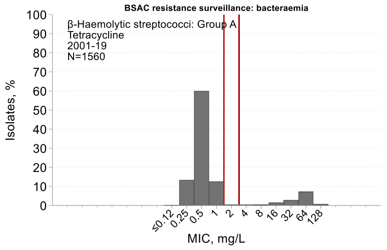

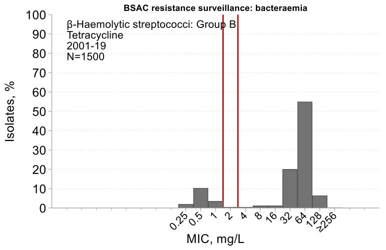

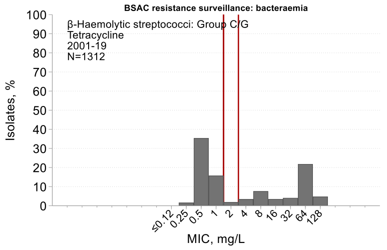

Tigecycline

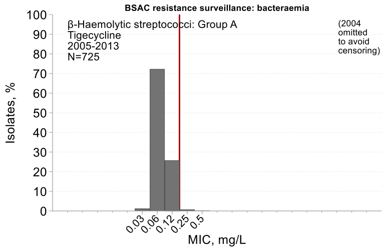

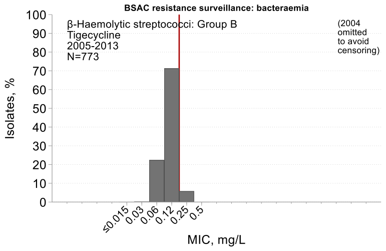

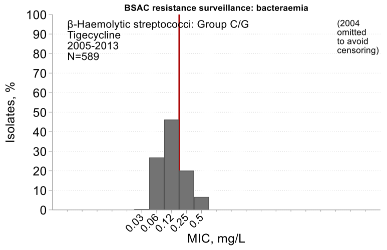

<table>
<tr><th colspan="3">β-Haemolytic streptococci</th></tr>
<tr><th>Group A</th><th>Group B</th><th>Group C/G</th></tr>
<tr>
<td>

Vancomycin

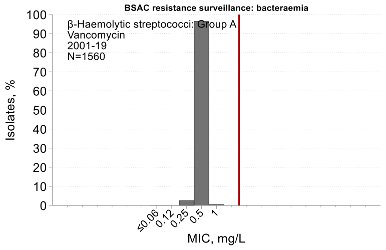

</td>
<td>

Vancomycin

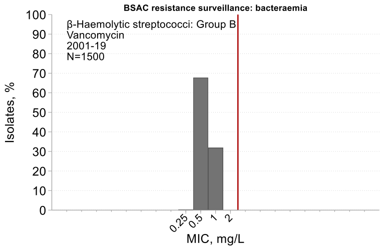

</td>
<td>

Vancomycin

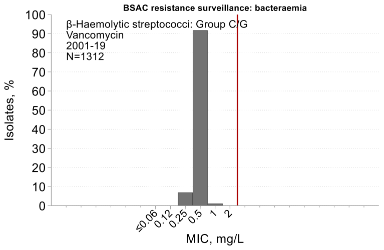

</td>
</tr>
</table>
